# Small Molecules Acting on Myofilaments as Treatments for Heart and Skeletal Muscle Diseases

**DOI:** 10.3390/ijms21249599

**Published:** 2020-12-16

**Authors:** Khulud Alsulami, Steven Marston

**Affiliations:** 1 Imperial Centre for Translational and Experimental Medicine, Cardiovascular Division, National Heart and Lung Institute, Imperial College London, London W12 0NN, UK; s.marston@imperial.ac.uk; 2 National Centre for Pharmaceutical Technology, King Abdulaziz City for Science and Technology, Riyadh 11461, Saudi Arabia

**Keywords:** contractility, sarcomere, cardiomyopathy, crossbridge cycle, therapeutics, drug trials

## Abstract

Hypertrophic cardiomyopathy (HCM) and dilated cardiomyopathy (DCM) are the most prevalent forms of the chronic and progressive pathological condition known as cardiomyopathy. These diseases have different aetiologies; however, they share the feature of haemodynamic abnormalities, which is mainly due to dysfunction in the contractile proteins that make up the contractile unit known as the sarcomere. To date, pharmacological treatment options are not disease-specific and rather focus on managing the symptoms, without addressing the disease mechanism. Earliest attempts at improving cardiac contractility by modulating the sarcomere indirectly (inotropes) resulted in unwanted effects. In contrast, targeting the sarcomere directly, aided by high-throughput screening systems, could identify small molecules with a superior therapeutic value in cardiac muscle disorders. Herein, an extensive literature review of 21 small molecules directed to five different targets was conducted. A simple scoring system was created to assess the suitability of small molecules for therapy by evaluating them in eight different criteria. Most of the compounds failed due to lack of target specificity or poor physicochemical properties. Six compounds stood out, showing a potential therapeutic value in HCM, DCM or heart failure (HF). Omecamtiv Mecarbil and Danicamtiv (myosin activators), Mavacamten, CK-274 and MYK-581 (myosin inhibitors) and AMG 594 (Ca^2+^-sensitiser) are all small molecules that allosterically modulate troponin or myosin. Omecamtiv Mecarbil showed limited efficacy in phase III GALACTIC-HF trial, while, results from phase III EXPLORER-HCM trial were recently published, indicating that Mavacamten reduced left ventricular outflow tract (LVOT) obstruction and diastolic dysfunction and improved the health status of patients with HCM. A novel category of small molecules known as “recouplers” was reported to target a phenomenon termed uncoupling commonly found in familial cardiomyopathies but has not progressed beyond preclinical work. In conclusion, the contractile apparatus is a promising target for new drug development.

## 1. Introduction

Cardiovascular diseases are a major cause of morbidity and mortality worldwide. In particular, cardiomyopathies and ischemic heart diseases are the most common causes of a chronic and progressive pathological condition termed heart failure [1,2]. Cardiomyopathy is a term that refers to abnormalities of heart muscle contractility, covering a heterogeneous range of aetiologies [3].

Dilated cardiomyopathy (DCM) is characterised by cardiac dilatation and impaired contractility (reduced ejection fraction and cardiac output), and 20–50% of cases are thought to be due to inherited mutations, linked to over 40 cardiac genes [4]. Heart failure with preserved ejection fraction (HFpEF) is a heterogeneous clinical syndrome, which in many patients is characterised by impairment of the left ventricle’s ability to relax and fill during diastole, resulting in insufficient blood flow to meet the body’s needs. HFpEF is estimated to affect approximately three million people in the US and is associated with significant morbidity and mortality. In both HFrEF and HFpEF, the primary abnormality is usually not in the contractile apparatus. Hypertrophic cardiomyopathy (HCM) is characterised by left ventricular hypertrophy and hypercontractility [5,6]. It is almost always caused by mutations of genes encoding sarcomeric proteins [5].

Current pharmacological treatment strategies of HCM, DCM and heart failure (HF) are mainly centred on managing the symptoms as well as minimising disease progression; however, these strategies are not disease-specific since they target neurohormonal system and excitation–contraction coupling while the basic disease mechanism remains untreated.

Cardiomyopathy is fundamentally due to abnormal contractility; therefore, targeting the contractile apparatus of cardiac muscle is critical. We now know enough about the mechanisms of contractility and its Ca^2+^-regulation and modulation by phosphorylation and mutations to be able to define suitable targets for drug treatments to alleviate the abnormalities of cardiomyopathies. The current focus of cardiac muscle studies is in the direction of developing new therapeutic approaches that act directly on the contractile apparatus or its regulators and thus, in theory, avoid many of the side effects of current treatments.

**Figure 1:**
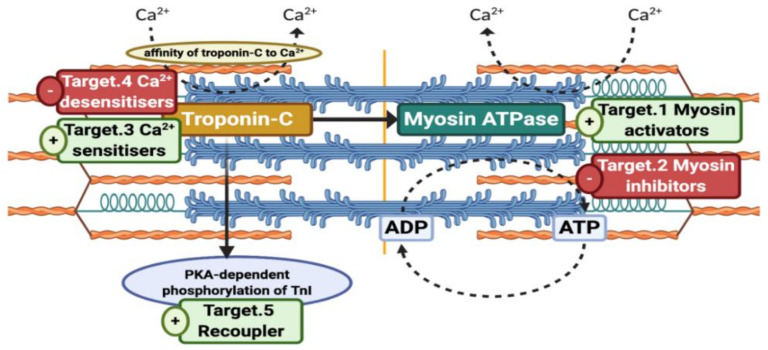
Five potential therapeutic targets in the contractile apparatus for small molecules. Original figure was made by using BioRender.com. The availability of myosin heads for interaction can be ameliorated via myosin activators or alleviated by myosin inhibitors. Similarly, affinity of troponin C towards Ca^2+^ ions can be increased (Ca^2+^-sensitisers) or decreased (Ca^2+^-desensitisers). Protein kinase A (PKA)-dependent phosphorylation of TnI was found to be lost in some forms of hypertrophic cardiomyopathy (HCM) and dilated cardiomyopathy (DCM), but it can be restored via Recouplers. Figure was created by using Biorender.com.

We have identified five classes of small molecule activity that have potential for various cardiomyopathies (see Figure 1). Hypertrophic cardiomyopathy (HCM) is manifested as hypercontractility of cardiac muscle and therefore should be targeted by myosin inhibitors or Ca^2+^-desensitisers [7,8]. A wider range of contractile abnormalities is found in myopathies characterised by inadequate cardiac muscle contractility such as HFrEF, HFpEF or DCM. In skeletal muscles, hypocontractile diseases occur as a result of mutations leading to congenital skeletal muscle myopathies [9]. In cardiac muscle, however, hypocontractility associated with heart failure is more complex in its nature and it is unlikely that we could target all its forms via a single compound. Currently, hypocontractility research is in the direction of myosin activation and Ca^2+^ sensitisation of thin filament and this may only be appropriate for a small range of cardiomyopathies. In this study, we have focused on the effects of small molecules on dilated cardiomyopathy (DCM), both familial and idiopathic. Lastly, there have been reports of a phenomenon termed Uncoupling that is associated with some cases of DCM and HCM and can be reversed by recoupling agents [10,11].

Already, some promising small molecules have been developed, such as the myosin activator Omecamtiv Mecarbil, to treat HF and DCM, and myosin inhibitor Mavacamten, to treat HCM. Both drugs have shown the viability of this approach of treatment for various forms of cardiomyopathy.

In this review, we investigate the potential therapeutic targets in the cardiac and skeletal muscle contractile apparatus and the actions of small molecules that act directly on contractile apparatus. We then offer an assessment of the advantages and disadvantages of these as treatments.

## 2. Contractile Activators as Treatments for Heart Failure and Muscular Myopathies

HFrEF is associated with structural or functional abnormalities of cardiomyocytes which, as a consequence, trigger neurohormonal axis activation and cardiac remodelling as compensatory mechanisms that ultimately result in chronic heart failure and death [12,13]. Current therapies, including β-blockers, angiotensin-converting enzyme (ACE) inhibitors, angiotensin receptor blockers (ARBs), mineralocorticoid receptor antagonists and angiotensin receptor–neprilysin inhibitors, act indirectly to perturb these compensatory mechanisms with a minimal capability to enhance cardiac function [14,15,16]. It has been hypothesised that direct activation of the contractile proteins would be a more effective treatment approach.

### 2.1. Cardiac Muscle Ca^2+^-Sensitisers (or Positive Inotropes)

Early attempts at developing cardiac agents were focused on finding compounds that can improve cardiac output identified as positive inotropes. Positive inotropes have been studied for decades and they can be categorised into either “calcium mobilisers”, which act by elevating the magnitude of calcium ions entering the myocytes or “calcium sensitisers”, which increase the sensitivity of myofilaments towards Ca^2+^ ions. It has been proposed that a successful inotropic drug would be one that increases contractility by directly increasing Ca^2+^ sensitivity independent of excitation–contraction coupling (EC-coupling) and adrenergic system. Ca^2+^-sensitisers that act upon troponin are considered as a subclass of inotropic agents. Several drugs with Ca^2+^ sensitising activity have been tested in the clinic over the last 30 years.

Levosimendan is a positive inotrope that can improve cardiac contractility without increasing oxygen demand of the myocardium unlike most inotropes [17,18]. Levosimendan binds to the hydrophobic patch of the N-domain of cardiac troponin C with EC_50_ of approximately 1 µM [19]. Unfortunately, levosimendan also has the ability to activate ATP sensitive K^+^ channel in addition to inhibiting III isoform of PDE enzyme [20]. Several large clinical trials of levosimendan in heart failure patients were conducted, summarised in Table 1. Although levosimendan appears to be more effective than dobutamine in acute situations it has not been found to be of value in long-term treatments.

Pimobendan is an oral Ca^2+^-sensitiser but also a PDE inhibitor developed by Boehringer Ingelheim Pharma KG. The pimobendan in congestive heart failure (PICO) trial in 1996 revealed that pimobendan contributed in increased risk of mortality compared to placebo (Table 1) [21,22]. Nowadays, pimobendan is approved only for the treatment of heart failure in dogs [23,24].

Bepridil is an oral Ca^2+^-sensitiser that is also a calmodulin (CaM) antagonist, Ca^2+^ channel blocker (negative inotrope) and potassium channel blocker. It is still marketed in US, Japan, Belgium, France and Ireland indicated for Angina pectoris [25]. Papadaki et al. showed that bepridil uncoupled troponin I phosphorylation from changes in Ca^2+^ sensitivity as well as enhancing Ca^2+^ sensitivity [10].

MCI-154 (Senazodan) is a PDE III inhibitor and Ca^2+^-sensitiser developed by Mitsubishi Pharma Corporation in Japan. It exerts its positive inotropic and chronotropic effects by binding directly to troponin C. Senazodan was tested in five trials in the 1990s and early 2000s, and in all of those trials, the small molecule showed favourable haemodynamic profile, as compared to dobutamine; nevertheless, no data were published thereafter about its development [26].

EMD57033 is a thiadiazinone derivative and a positive inotrope that increases the force of the contraction without altering intracellular the Ca^2+^ transient and has minimal PDE inhibition activity [27]. EMD57033 acts as a Ca^2+^-sensitiser by binding to the hydrophobic pocket of the C-domain of troponin C leading to a weak cTnC–TnI interaction [18]. Baudenbacher et al. showed the left shift in log Ca^2+^ vs. relative force in mice was associated with a higher susceptibility to ventricular arrhythmias [28]. As a “pure” Ca^2+^-sensitiser, EMD57033 ought to be a superior inotrope but due to bioavailability problems, it has not been tested in the clinic.

AMG 594 is an allosteric cardiac troponin activator that is claimed to be direct and specific, developed by Cytokinetics for the treatment of HF [29]. Conference proceedings reported that AMG 594 acts solely on the sarcomere by sensitising cardiac troponin to Ca^2+^ ions, leading to more myosin heads engaging with actin filaments, and more contractile force being generated [30]. A phase I clinical trial of AMG 594 was completed on August 2020, but there are no available data so far.

Ca^2+^-sensitisers that act upon troponin are considered as subclass of inotropic agents. In principle, developing a “pure” Ca^2+^-sensitiser that can enhance cardiac contractility without affecting EC-coupling or compromising cardiac energetics would avoid most of the defects of current compounds. In clinical practice, bepridil, pimobendan, MCI-154 and levosimendan have failed as treatments for chronic HF largely because of the off-target effects of such drugs, notably PDE inhibition and enhanced arrhythmia [28,31].

The only well-researched apparently pure Ca^2+^-sensitiser is EMD57033 with preclinical data, suggesting that the concept of troponin Ca^2+^ sensitisation may be viable; however, its bioavailability problems have prevented any clinical studies. The new troponin activator developed by Cytokinetics (AMG 594) claims to be a “pure” and selective cardiac troponin activator, although little data have been published, and phase I clinical trial has only recently completed. According to the preclinical data, AMG 594 seems to be enhancing cardiac contractility independently of EC coupling in a similar way to Omecamtiv [30]. It should be noted that myofilament Ca^2+^ sensitisation by any route has the potential to enhance arrhythmias which may limit the clinical utilisation of any Ca^2+^-sensitiser [28].

### 2.2. Cardiac Myosin Activators

The development of direct myosin activators was fuelled by hypothesising that direct activation of cardiac sarcomere can improve cardiac performance with an additional advantage of being independent of the usual neurohormonal response and cardiac remodelling [35,36]. Selective activation of the actin and myosin interaction aims to avoid disadvantages of classic inotropic agents that enhance cardiac contractility but, at the same time, increase oxygen demand, heart rate and intracellular calcium transient which are linked to hypotension, arrhythmias and mortality [31,33,37,38].

#### 2.2.1. Omecamtiv Mecarbil

A high-throughput screening (HTS) of around 40,000 small molecules, using a myofibrillar ATPase screen, led to the discovery of Omecamtiv Mecarbil (OM) [36,39]. OM (formerly known as CK-1827452 and AMG-423) was developed by Cytokinetics in collaboration with Amgen as a novel, allosteric cardiac myosin activator. The molecular mechanism initially proposed by the Cytokinetics group was that OM binds the catalytic S1 domain of the myosin, causing four-fold acceleration of the phosphate (Pi) releasing step thus, enhancing duty ratio [35,40]. However, once the compound became available to third party researchers several inconsistencies in the model were found. Nagy et al. pointed out that OM increased the Ca^2+^ sensitivity at concentrations of 0.1 μM and higher in permeabilized rodent cardiomyocytes. Furthermore, activation was biphasic and concentrations above 1 μM, OM inhibited force production [41]. Moreover, Liu et al. demonstrated that OM reduced the velocity of crossbridge cycles in an in vitro motility assay, using porcine fibres [42].

The molecular mechanism was explained by Woody et al.; they showed that OM caused a 10-fold reduction in the size of the working stroke (from 5.4 nm to around 0 at 10 μM) as well as five-fold prolongation in the actomyosin attachment duration [43]. These observations account for the inhibitory and Ca^2+^-sensitising effect of OM by being a cooperative activator of the thin filament [43,44]. All in all, it is clear now that OM exerts its action by recruiting more crossbridge cycles instead of altering their dynamics which can be described as “more hands pulling on the rope”.

A preclinical model of pigs with left ventricular dysfunction showed that treatment with OM contributes to elevated myocardial O_2_ consumption [45]. It has been argued that the elevation of myocardial O_2_ demand, a sign of cardiac ischemia, was due to the administration of high concentrations of OM [46]. Similarly, Teerlink et al. reported in their first-in-man trial that signs of myocardial ischemia emerged at plasma concentrations above 1200 ng/mL [47]. This is a negative property of OM as it contributes to narrow its therapeutic window.

There have been several clinical trials of OM that are detailed in Table 2. OM has been studied in healthy men, patients with chronic systolic HF and patients with acute HF. Phase I trials were conducted in healthy participants to assess pharmacokinetics and pharmacodynamics of OM in intravenous and oral formulations. So far, OM has been well-tolerated and elevation in ejection fraction and cardiac output was observed. However, in one of these trials (ATOMIC-HF) the primary (relief of dyspnoea) and secondary endpoints were not met which has led some to question the value of further trials [48,49]. Recently, results of a large randomised, placebo-controlled phase III Global Approach to Lowering Adverse Cardiac Outcomes through Improving Contractility in Heart Failure (GALACTIC-HF) trial were published (see Table 2) [50]. The primary outcome was a composite of a heart-failure event or cardiovascular death (whichever comes first). Secondary outcomes included cardiovascular death, change in in the total symptom score on the Kansas City Cardiomyopathy Questionnaire (KCCQ) from baseline to week 24, first hospitalisation for heart failure or death. A significant yet modest reduction in the incidence of the composite primary outcome was shown in 37% of the OM group and in 39.1% of the placebo group (95% confidence interval (CI), 0.86 to 0.99; *p* = 0.03). Moreover, the trial did not show any significant improvement in the secondary outcomes and the incidences of myocardial ischemia, ventricular arrhythmias and death were similar in both OM and placebo groups. It is worth noting that a higher treatment benefit was suggested in patients with LVEF of 28% or less (New York Heart Association (NYHA) class of III or IV).

#### 2.2.2. Danicamtiv

Danicamtiv (formerly known as MYK-491) is the lead candidate myosin activator in a programme developed by MyoKardia (now sold to Bristol Meyers Squibb) in collaboration with Sanofi for the treatment of systolic heart failure, and specifically DCM. The small molecule is catching up with OM and currently just finished a phase II clinical trial. MyoKardia claims that “MYK-491 directly activates cardiac actomyosin, enhancing the initial step of the force-producing chemo-mechanical cycle by Increases the rate of Pi release and availability of myosin-heads”; studies on the molecular mechanism have not been published but it is suggested that the mechanism is similar to OM [51,52].

Danicamtiv binds selectively to human cardiac myosin isoform without binding to skeletal or smooth muscle isoforms resulting in the elevation of ATPase turnover rate (+85% in ventricular myofibrils) and increased Ca^2+^ sensitivity (+0.35 pCa unit) [53]. In acutely treated male beagle dogs with induced HF (*n* = 7), administration of Danicamtiv prolonged SET and improved LVEF in addition to cardiac output [53].

Danicamtiv has been studied in a single-ascending dose phase IIa trial in 40 patients with chronic and stable HFrEF (see Table 2). The small molecule caused a dose-dependent elevation in left ventricular stroke volume (LVSV) and an increase in SET in medium and high concentrations. No changes in diastolic blood pressure or heart rate were reported except for a minor reduction systolic blood pressure in high concentration cohort. Moreover, no signs of cardiac ischemia were reported at the doses used [53]. Most of these results are rather similar to OM; however, the effects of chronic treatment have not been studied.

#### 2.2.3. EMD57033

EMD57033 has also been proposed as a myosin activator, in addition to being a positive inotrope and Ca^2+^-sensitiser acting on cardiac troponin [27]. However, a recent study shows that EMD57033 is an effective preserver of myosin activity rather than an activator [27,61].

#### 2.2.4. Assessment of Myosin Activators

Investigating the literature led to finding reports on three compounds that may have cardiac myosin activation properties, and only two of those compounds (Omecamtiv Mecarbil and Danicamtiv) made it to the advanced stages of drug development, with the first in phase III and the latter catching up. Both Omecamtiv Mecarbil and Danicamtiv have been formulated in oral and intravenous dosage which is necessary for further drug development.

Despite the encouraging results of Omecamtiv Mecarbil as a myosin activator in vitro and as a contractile activator in vivo in animal studies, the small molecule shows a limited efficacy in clinical trials [48,56]. It has been questioned whether continued trials with Omecamtiv Mecarbil are worthwhile [49]. Nevertheless, Omecamtiv Mecarbil has been granted fast track designation as a potential new treatment for heart failure from the US Food and Drug Administration (FDA) [62]. With the limited data so far, it appears that Danicamtiv acts in the same way as OM.

Current therapeutic options for the treatment of HFrEF have been successful in reducing mortality rates which sets the bar high for any new therapy. All available treatments for HFrEF are focused on reducing the load on the heart, thus preserving the heart function only without improving its mechanical output. Myosin activators have the advantage of acting directly on the contractile apparatus to enhance cardiac contractility by prolonging systolic ejection time (SET), a property that cannot be found in the conventional positive inotropes. The question remains, will direct activation of the contractile apparatus be of benefit for a long-term treatment of chronic HF? Phase III GALACTIC-HF clinical trial was conducted for around 22 months and showed a minor reduction in the incidence of a composite of HF-event or death due to cardiovascular causes. With such results it seems that myosin activators (OM specifically) are unlikely replacing current standard therapies any time soon.

Although myosin activators increase contractility in a selective manner which can be valuable in treating systolic heart failure, their Ca^2+^ sensitising effect, which might promote diastolic dysfunction and arrhythmias, limits the expectations of their future therapeutic worth. This is an inevitable consequence of the cooperative allosteric mechanism of regulation of contractility that the compounds act upon. This cooperative allosteric mechanism is likely to be responsible for the diastolic dysfunction observed at high doses that could limit the therapeutically safe range of doses. Indeed, a narrow therapeutic window is implied by the need for dose titration in most clinical studies.

In most aspects, the effect of OM mimics the effect of mutations that cause hypertrophic cardiomyopathy. In particular, both increase myofilament Ca^2+^-sensitivity by similar amounts leading to hypercontractility at the expense of possible diastolic dysfunction and enhanced arrhythmia [7,35,43].

In their book *Therapeutic strategies for managing heart failure* (2000), Silber and Katz described the failing heart as “a sick, tired horse pulling a wagon up a steep hill”, which is an excellent analogy to view heart failure and its therapeutic options for helping the horse and wagon “up the hill” [63]. The currently used therapies are mostly based on “unloading the wagon” as their mode of action whilst myosin activators are thought to be “whipping the horse”, which may not be therapeutically advantageous for long-term treatment.

Omecamtiv Mecarbil has been studied in a spectrum of aetiologies of HF, unlike Danicamtiv, which has been promoted as potential therapy for DCM. This raises the question, would a more precise target such as familial DCM, especially if caused by mutations in contractile proteins, lead to better and more successful outcomes? At the same time, would it be worthwhile to target such a small fraction of HFrEF patients?

### 2.3. Skeletal Muscle Activators and Ca^2+^-sensitisers

Skeletal muscle congenital myopathies are characterised by muscle weakness. They can be due to abnormalities of the contractile apparatus or a reduction in the density of muscle innervation, the rate of neuromuscular junction activation or the efficiency of synaptic transmission. It was proposed that a small-molecule fast-skeletal–troponin activator, would increase muscle strength in myopathy due to mutations in contractile proteins but could also be useful by amplifying the response of muscle when neural input is otherwise diminished secondary to neuromuscular disease [64]. This is possible because, unlike cardiac muscle, skeletal muscle has the advantage of being regenerative thus muscle activation can promote muscle growth [65]. A screen for Ca^2+^ sensitising agents that target fast skeletal muscle troponin by Cytokinetics yielded the following compounds: Tirasemtiv (or CK-2017357), Reldesemtiv (or CK-2127107) and CK-2066260.

#### 2.3.1. Tirasemtiv

Tirasemtiv (or CK-2017357) was developed by Cytokinetics as an orally administered, highly specific small molecule fast skeletal troponin activator (FSTA) with affinity of 40 nM. Russell et al. proposed that amplifying the sarcomeric response to insufficient neuronal input by increasing Ca^2+^ sensitivity to troponin-tropomyosin complex can improve muscular force generation and physical performance in patients with neuromuscular disorders such as Myasthenia gravis and amyotrophic lateral sclerosis (ALS). In a passive transfer experimental autoimmune myasthenia gravis (PT-EAMG) rat model, Tirasemtiv increased the force of muscle contraction at submaximal nerve stimulation frequencies, increased grip strength, and decreased muscle fatigability [64].

Hansen et al. reported that Tirasemtiv augmented the skeletal muscle response to nerve input in healthy human males, in a randomised, double-blind, four-period crossover study [66]. Tirasemtiv underwent phase II clinical trials in patients with ALS (ClinicaltrialNCT01709149), peripheral vascular disease (NCT011310313) and myasthenia gravis (NCT01268280). The ability of Tirasemtiv to pass the blood–brain barrier (BBB) contributed in adverse events such as dizziness and fatigue.

Nevertheless, in 2012, Tirasemtiv was granted fast track designation from the American FDA in addition to the orphan drug status for ALS in Europe and the US [67]. Despite all that, in 2017, Cytokinetics decided to suspend the development of Tirasemtiv due to the negative results from the VITALITY-ALS trial [68].

#### 2.3.2. Reldesemtiv

Reldesemtiv (formerly known as CK-2127107 or CK-107) is a next generation, orally available FSTA developed also by Cytokinetics in collaboration with Astellas Pharma with a potential benefit in improving skeletal muscle function and physical performance in neuromuscular disorders such as spinal muscular atrophy (SMA) and amyotrophic lateral sclerosis (ALS), in addition to muscle fatigue in chronic obstructive pulmonary disease (COPD) [69]. In phase I clinical trials in healthy human, Reldesemtiv showed significant elevation in tibialis anterior muscular response in a dose-dependent fashion that appears to be superior to Tirasemtiv [70]. Results from FORTITUDE-ALS trial indicated that there was no statistical significance in its primary endpoint which is change from baseline in slow vital capacity (SVC) and after 12 weeks of dosing based on a pre-specified dose-response relationship (*p* = 0.11) [71].

CK-2066260 is also a Tirasemtiv-structural analogue FSTA developed by Cytokinetics, in collaboration with Astellas, as a part of skeletal muscle activator research programme. Unlike Tirasemtiv and Reldesemtiv, CK-2066260 has been tested on nemaline myopathy patients with nebulin mutations [72,73].

These newly developed skeletal muscle troponin-specific Ca^2+^-sensitisers provide an interesting comparison with cardiac Ca^2+^-sensitisers. Skeletal muscle is resistant to arrhythmia and is also capable of regenerating; therefore, Ca^2+^-sensitisers are safer and also can stimulate nerve and muscle growth thus potentially alleviating a wide range of neuromuscular disorders. Nevertheless, preclinical potential has not translated into successful trials yet as the lead compound, Tirasemtiv, has been suspended and its analogue Reldesemtiv failed in meeting its endpoints in the FORTITUDE-ALS trial (Table 3).

#### 2.3.3. Piperine

Nogara et al. suggested that myosin activation of fast skeletal muscles can be of therapeutic benefit in treating obesity and type 2 diabetes. Transferring myosin heads from the super-relaxed (SRX) to the disordered-relaxed (DRX) state can increase the metabolic rate of the whole human body by 2–4 MJ per day [74,75]. Additionally, Nogara et al’s study found a fluorescent probe on regulatory light chain (RLC) of myosin that shows shorter wavelengths upon the transition from the SRX to the DRX. This was used for a high throughput screening a library of over 600 compounds which resulted in identifying Piperine a naturally occurring alkaloid extracted from black pepper as a suitable candidate [74,76].

As a cardiac myosin activator, Piperine fails due to the lack of specificity for skeletal muscle and off target effects. It is conceivable that further development from piperine targeted at cardiac muscle SRX destabilisers may uncover a useful compound.

## 3. Contractile Inhibitors as a Treatment for Hypertrophic Cardiomyopathy (HCM)

Hypertrophic cardiomyopathy is a hypercontractile disease and the majority of known mutations that cause HCM are in thick filament proteins, myosin and MyBP-C [82,83]. It is, therefore, valuable to find small molecules that inhibit cardiac myosin in the contractile apparatus for targeted treatment HCM [84,85,86].

HCM can be divided into two categories: obstructive hypertrophic cardiomyopathy (HOCM or oHCM), in which the left ventricular outflow tract (LVOT) is obstructed; or non-obstructive hypertrophic cardiomyopathy (nHCM), which is characterised by the absence of LVOT at rest (<30 mm Hg) [87]. Pharmacological treatment options for patients with HOCM and nHCM include non-vasodilating β-receptor blockers titrated to maximum tolerated dose, anti-arrhythmic drug disopyramide as an add-on treatment (for HOCM) or non-dihydropyridine calcium channel blockers [88]. The current therapeutic options lack specificity and they have modest efficacy in controlling LVOT gradients as they do not target the main cause for the disease which is the hypercontracting sarcomere.

### 3.1. Ca^2+^-Desensitisers

Ca^2+^-desensitisers that act on troponin are a group of small molecules that, theoretically, aim to treat HCM by desensitising the thin filament toward Ca^2+^ ions, thus reducing contractility.

#### 3.1.1. Green Tea Catechins (EGCg and ECg)

Consumption of green tea has been linked to a lower risk of cardiovascular diseases in several studies mainly due to the presence of biologically active compounds in green tea are the polyphenols known as Catechins [89,90,91]. Epigallocatechin-3-gallate (EGCg) is the most widely studied catechin which has been reported as a Ca^2+^-desensitiser [92]. Tadano et al. also reported that epicatechin gallate (ECg) shares the direct Ca^2+^ desensitisation property with EGCg through binding to troponin C. In skinned cardiac muscle fibres, EGCg showed a greater desensitisation effect than ECg and cardiac-selective molecular action. In isolated working hearts of an HCM mouse model with increased Ca^2+^ sensitivity, EGCg restored cardiac output by improving diastolic dysfunction suggesting a potential therapeutic benefit in HCM. When applied to isolated cardiomyocytes of guinea pig HCM model, EGCg showed a poor potency as it requires 30–100 µM to desensitise the myofilament, while at lower concentrations (<1 µM), significant off-target effects were observed [93].

#### 3.1.2. Nebivolol

Nebivolol is a β-adrenergic receptor antagonist that also has a Ca^2+^ desensitisation activity at EC_50_ of 10 μM [94,95]. Nebivolol selectively desensitised permeabilised cardiac muscle from Mybpc3-targeted knock-in (KI) cardiomyopathy mouse model without resulting in any significant change in contractility [95]. The drug also had a negative impact on shortening in the cardiomyocyte, while causing a slower contraction and relaxation [96].

Troponin based Ca^2+^-desensitisers have not been researched much and have not gone beyond in vitro studies for various reasons. EGCg and related compounds are known to act promiscuously in vivo with multiple actions that preclude their use outside in vitro situations and no therapeutic benefit should be anticipated [97,98,99]. Nebivolol, on the other hand, seems to be a more promising desensitiser as it, so far, ticked all the boxes needed in a good desensitiser. The small molecule is cardio-specific that effectively worked in animal models and already approved as a β-blocker, accordingly, it has an established safety profile. Since β-blockers are currently the first line of treatment in HCM; therefore, having a compound that acts as β-blocker and Ca^2+^-desensitiser may act synergistically to treat HCM.

### 3.2. Recouplers

Protein kinase A (PKA)-mediated phosphorylation of MyBP-C and troponin I modulate the Ca^2+^ switch within the contractile apparatus. The absence of modulation of Ca^2+^ sensitivity by troponin I phosphorylation results in blunted response to adrenergic stimulation in a phenomenon known as uncoupling. In familial cardiomyopathies, mutations in thin filament proteins often associated with the loss of this modulation, thus uncoupling is postulated to contribute to the HCM or DCM phenotype [10,11,100,101]. In support of this, in vivo transgenic mouse studies showed that uncoupling leads to heart failure under stress [102].

Recouplers are a novel category of small molecules that demonstrated the ability to reverse the uncoupling of troponin I phosphorylation and Ca^2+^ sensitivity. Compounds that are shown to be effective as recouplers, to date, include EGCg, Silybin B, Dehydrosilybin B, resveratrol, novobiocin [97]. As compounds under this category are in early stages of research, our assessment system seemed inapplicable.

### 3.3. Myosin Inhibitors

The concept of utilising myosin inhibitors in biochemical and physiological studies has been around for a long time. Early myosin II inhibitors include of 2,3-butanedione monoimine (BDM), *N*-benzyl-*p*-toluene sulphonamide (BTS) and blebbistatin; of these, only blebbistatin has been considered as a drug prototype [103,104,105,106]. Recently a new range of unrelated cardiac muscle specific myosin inhibitors has been developed as potential treatment for HCM.

#### 3.3.1. Blebbistatin and Its Analogues

Blebbistatin is a myosin II inhibitor that has been widely used as a research tool in various areas such as muscle physiology, cancer, cell migration and differentiation [107]. Structural and functional studies showed that the inhibitory effect of blebbistatin is due its ability to stabilise myosin heads of the thin filament in SRX thus decreasing the number of active force producing myosin heads [108].

The majority of myosin II isoforms are inhibited by blebbistatin with the highest affinity to skeletal muscle myosin II (EC_50_ 0.1–5 mM) and intermediate affinity for cardiac and non-muscle myosin II isoforms (EC_50_ 1–10 mM) ruling out blebbistatin as a cardiac compound [107]. Multiple derivatives of blebbistatin have been developed in order to improve its physicochemical and pharmacological properties [109], but improved specificity has not been achieved yet.

#### 3.3.2. Mavacamten

The journey of developing Mavacamten as a small molecule for treatment of HCM started with a hypothesis that excess sarcomere power can be the primary cause of HCM and thus, the pathological phenotype of HCM could be alleviated by normalising the hyperdynamic sarcomeric power [110,111,112]. A chemical screening for compounds with the ability to reduce actin-activated ATPase rate of myosin by MyoKardia yielded MYK-461 or “Mavacamten”.

Transient kinetic analyses showed that Mavacamten decreases the rate of inorganic phosphate (Pi) release, the rate-limiting step of the chemomechanical cycle without altering the rate of ADP release in actin-activated state. Mavacamten binds to myosin where it stabilises the super-relaxed (SRX) conformation [113,114]. In mouse cardiac and bovine myofibrils, treatment with Mavacamten showed that Mavacamten reduced ATPase activity (EC_50_ 0.3 μM in mouse) [112]. Similarly, a dose-dependent reduction in fractional shortening (FS) without affecting calcium transient was observed in isolated rat cardiomyocytes (EC_50_ 0.18 μM) [112].

In vivo effects of long term Mavacamten treatment were investigated in mouse models of HCM expressing α-cardiac myosin heavy chain mutations and showed diminution of fibrosis and myocyte disarray [112]. A Feline model of HCM showed that IV treatment with Mavacamten resulted in reduction of cardiac contractility, indicated by reduced fractional shortening (*p* = 0.01), and left ventricular outflow tract (LVOT) pressure gradient (*p* = 0.0007) [115]. Similarly, Del Rio et al. assessed acute and chronic cardiac responses of dogs to Mavacamten. In acute studies, Mavacamten reduced inotropic indices (*p*-value < 0.05) whilst maintaining systemic pressure (MBP: 107 ± 6 to 109 ± 5 mmHg) [116].

Pharmacokinetic properties were extensively studied by Grillo et al. showing that Mavacamten can be administered via both oral and intravenous routes. Notably, Mavacamten has a high volume of distribution (V_d_ = 9.5 L/kg), plasma clearance of 0.51 mL/min/kg and half-life (t_1/2_) of 9 days [117].

Clinical trials on Mavacamten are detailed in Table 4. Earlier clinical trials (PIONEER-HCM and PIONEER-OLE) targeted patients with HOCM, followed by MAVERICK-HCM, a trial designed for patients with nHCM. In the PIONEER-HCM trial, Mavacamten was well-tolerated in the two cohorts of patients with HOCM [118]. Moreover, PIONEER-OLE is an open-label extension trial in patients from PIONEER-HCM and it showed reduced LVOT obstruction and improve exercise capacity without cardiac-related adverse events [119]. MAVERICK-HCM, on the other hand, was conducted on patients with nHCM and was designed to evaluate the dosing and safety of Mavacamten. Adverse events reported in the trial in 90% of Mavacamten group while in 68% in the placebo group [120]. The most commonly reported adverse events were palpitations, dizziness and fatigue [120]. Moreover, the trial showed reduction in cardiac markers *N*-terminal pro b-type natriuretic peptide (NT-proBNP) and Cardiac troponin I (cTnI) [120].

The results of phase III clinical trial (EXPLORER-HCM) were presented in European Society of Cardiology virtual congress on August 2020 [121]. EXPLORER-HCM was the largest placebo-controlled randomised clinical trial in HCM with 251 patients from 13 countries. The primary endpoint was an elevation in peak oxygen consumption (pVO_2_) by 1.5 mL/kg per min or greater and at least one NYHA class reduction OR a 3.0 mL/kg per min or greater pVO_2_ increase without NYHA class worsening [122]. The composite primary endpoint was met in 37% of Mavacamten group versus 17% of the placebo group (*p* = 0.0005). Moreover, complete abolition of all LVOT gradients (resting and post-exercise) was achieved in 57% patients in Mavacamten group. In general, Mavacamten was associated with improvement in exercise capacity, LVOT obstruction and NYHA functional classification with reduction in plasma NT-proBNP and cTnI.

Further developments by MyoKardia have yielded compounds that may have a better pharmacological profile than Mavacamten. Preclinical pharmacodynamics data of MYK-581 were presented at the American Heart Association Scientific Sessions 2019 [123]. MYK-224 is another new Mavacamten analogue. There are no available preclinical data on MYK-224 besides what is stated on MyoKardia’s website [124]. A Phase I clinical trial of MYK-224 has been initiated to assess safety, tolerability and pharmacokinetics of MYK-224 in healthy participants. The trial was suspended on May 2020 in response to the COVID-19 pandemic and resumed recruitment again in August 2020 [125].

#### 3.3.3. CK-3773274 (or CK-274)

CK-274 is described as a next generation, oral cardiac myosin inhibitor developed by Cytokinetics to treat HCM. Its mechanism is not detailed but is likely to be the same as Mavacamten. CK-274 was studied in bovine cardiac myofibrils, using the ATPase assay as described in Malik et al., for OM; which resulted in identifying a cardiac-specific inhibitory effect (EC_50_ 1.26 μM) with little effect on Ca^2+^-sensitivity [35,137,138]. Using healthy male Sprague Dawley (SD) rats, cardiac contractility was assessed in vivo via single oral doses ranging from 0.5 to 4 mg/kg at multiple time points after administering the single dose [138]. Echocardiography results indicated that CK-274 caused a FS reduction in a dose-dependent manner that peaked at 1 hour. CK-274 decreased FS in a similar manner in both WT and R403Q (HCM mutation) transgenic mice [139]. Currently, CK-274 is under investigation in REDWOOD-HCM trial (Table 4) for patients with HOCM which has been temporarily suspended due to COVID-19 pandemic [140]. All available data come from unpublished conference proceedings.

#### 3.3.4. Assessment of Cardiac Myosin Inhibitors

Blebbistatin is the prototype direct myosin inhibitor and further investigation led to identifying 5 compounds with therapeutic potential in cardiac muscle. There are other small molecules with myosin inhibition properties excluded, such as BTS, BDM and others as they only target myosin isoforms that are found in fast skeletal muscle fibres or due to poor physicochemical and pharmacological properties.

Compared to Omecamtiv Mecarbil, Mavacamten seems to be developing in the process of drug development faster with more favourable results. In particular, the effect of Mavacamten especially in HOCM seems to be largely independent of HCM genotype which is valuable in a heterogeneous disease such as HCM [121,141]. The reason for the success of Mavacamten vs. OM is most likely related to the disease targeted. The abnormality in HCM is well understood and confined to the sarcomere and stabilising the SRX presents a specific mechanism, whereas the abnormalities targeted by OM are much more diffuse.

The positive results of PIONEER-HCM, MAVERICK-HCM and EXLORER-HCM show Mavacamten to be an effective treatment for nHCM and HOCM in patients with a mean age of around 50. Additionally, Mavacamten could be especially beneficial in younger patients to minimise cardiac remodelling and avoid invasive surgical interventions. Moreover, it has been hypothesised that Mavacamten could be valuable as a long-term sole treatment without the need for β-blockers and calcium channel blockers. Two clinical trials are currently progressing (see Table 4). VALOR-HCM is an ongoing trial that will investigate the impact of Mavacamten in younger HOCM patients who are eligible for septal reduction therapy, while MAVA- LTE trial aims to study the long-term effect of Mavacamten for up to five years.

The only downside of Mavacamten is that it has less favourable pharmacokinetic profile which include long t1⁄2 (≈9 days) and low plasma clearance rate (≈0.51 mL/min/kg) [117]. CK-274 has a much shorter half-life (about 12 h) and MyoKardia has recently developed multiple Mavacamten analogues such as MYK-581 and MYK-224 for shorter half-life which can reduce the time necessary to achieve steady-state concentration.

## 4. Discussion

**Figure 2:**
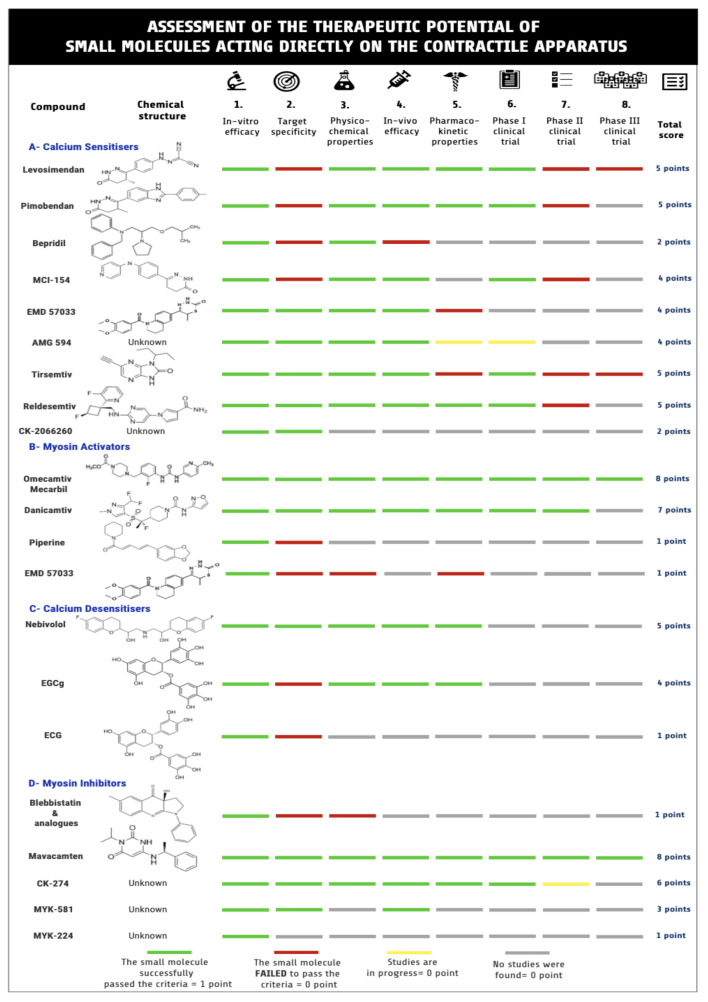
Assessment of therapeutic potential of small molecules that act on the contractile apparatus. Searches of the literature yielded 21 compounds worth further consideration. The compounds were evaluated and scored according to eight different criteria summarised in the figure. Created by using Venngage infographic maker.

In this review, the contractile apparatus of cardiac and skeletal muscle and the small molecules that can target it were investigated, with the objective of identifying the proper small molecule to treat muscle diseases (Figure 2). In the past decade, interest in developing small molecules that can act directly on the contractile apparatus have emerged. This phase of research was suggested as the “fourth wave” of muscle research [65].

### 4.1. Targets within the Contractile Apparatus

**Figure 3:**
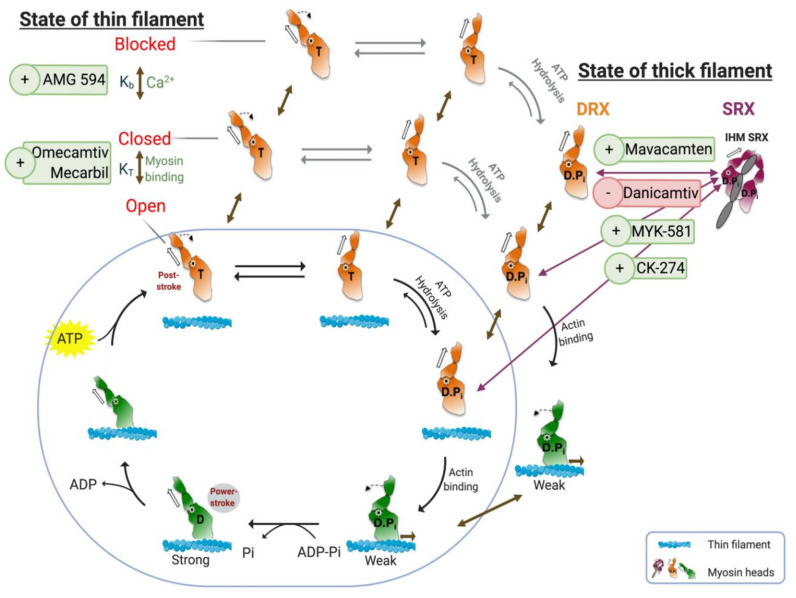
The chemomechanical crossbridge cycle and its regulation via troponin–tropomyosin (thin filament state) and super-relaxed/disordered-relaxed (SRX/DRX) equilibrium. The crossbridge is represented in the blue circle. The availability of actin-binding sites is regulated by the state of thin filament (top left). The equilibrium between blocked (no myosin bound) and closed (weak myosin-binding) is controlled by Ca^2+^. Myosin heads regulate the closed-open state in a cooperative fashion. Only thin filament in open state can participate in the chemomechanical cycle. Two small molecules that interact with both transitions are illustrated. The availability of myosin heads is regulated by the SRX/DRX equilibrium, and only myosin heads in DRX can be part of the crossbridge cycle. Four small molecules can regulate the transition, as shown. Figure was created by using Biorender.com as a modified version from References [65,83].

The targets we have defined do not act on the crossbridge cycle; instead, they interfere with the cycle magnitude by “modulating its modulators”, as illustrated in Figure 3. As myopathies are due to either hypercontracting or hypocontracting muscle, modulating the sarcomere at the troponin or myosin level might be more effective in treating cardiac muscle disorders. In case of HCM, a classic cardiac muscle gain-of-function disorder, myosin inhibitors and Ca^2+^-desensitisers address the defect directly. Experimentally, myosin inhibitors seem promising while developing the appropriate Ca^2+^-desensitisers appears to be more challenging. In contrast, HFrEF and DCM have more complex aetiologies. Compensatory mechanisms such as neurohormonal axis activation and cardiac remodelling (i.e., fibrosis and inflammation) are often associated with HFrEF and DCM leading to chronic heart failure [12,13]. Heart failure also involves abnormalities in myocardial metabolism which can trigger systemic metabolic changes [142]. Nevertheless, enhancing contractility directly via myosin activation and Ca^2+^ sensitisation have been proposed as a possible approach, using small molecules with better pharmacological profiles than existing inotropes.

### 4.2. Selected Small Molecules with Potential Therapeutic Value

Out of 21 compounds investigated, only six compounds showed any potential to be of therapeutic value. Omecamtiv Mecarbil and Danicamtiv (myosin activators), Mavacamten, CK-274 and MYK-581 (myosin inhibitors) and lastly AMG 594 (Ca^2+^-sensitiser) are small molecules that act allosterically to correct cardiac muscle abnormalities. OM acts by allosterically recruiting more crossbridge cycles and just recently completed phase III GALACTIC-HF clinical trial as a treatment for HF. Danicamtiv is another myosin activator with what appears to be a similar mode of action as OM; however, it is targeted more narrowly to DCM as a form of HF. Mavacamten is a first-in-class myosin inhibitor that showed clinical benefit in patients with HCM proved by the recently published results from phase III EXPLORER-HCM trial. CK-274 is another myosin inhibitor developed by Cytokinetics and currently in phase II trials. MYK-581 is a Mavacamten analogue developed with the aim of improving pharmacokinetic properties. AMG 594 is a novel Ca^2+^-sensitiser and troponin activator suggesting a therapeutic benefit in HF.

### 4.3. Limitations and Difficulties

The scarcity of published peer-reviewed data on some of the compounds in their early stages of development is a major problem for making a critical evaluation of the small molecules. What we were able to obtain came from the companies in the form of abstracts, posters, press releases or presentations at private meetings. As all of these forms of literature are unpublished and non-peer-reviewed, they were largely uninformative with the possibility of bias. Another problem is that the structure of the new molecules is not always published and that the small molecules themselves are not necessarily available to third parties for investigation. This is important for basic research; the best example for that is the molecular mechanism of action of OM which was not worked out until the drug was available to third parties and was quite different from Cytokinetics’s original proposed mechanism [35,43].

### 4.4. Future Prospects

In the case of myosin and troponin activators in cardiac muscle disorders, clinical trials indicated moderate improvement in cardiac functions; however, whether they will be able to replace the current therapies remains unlikely. In contrast, clinical trials showed that myosin inhibitors, as exemplified by Mavacamten, are promising small molecules in treating HOCM. Its success raises the possibility that myosin inhibitors could be a curative therapy for HOCM and it is now also being proposed as “a unique and precise therapy, to our non-obstructive HCM patients and potentially other similar individuals suffering from HFpEF” [143]. The subgroup identified for future evaluation of Mavacamten is estimated to include approximately 10–20% of the broader HFpEF population.

## Figures and Tables

**Table 1:** Overview of reported clinical trials on calcium sensitisers.

Drug Name	Trial and Year(s)	Study Design	(*n*) Targeted Population	Aim	Key Findings	Ref.
**Pimobendan**	PICO 1996	Randomised, double blind, placebo controlled trial	(317) Patients with LVEF ≤ 45%	To determine the effects of pimobendan 2.5 and 5 mg daily on exercise capacity in patients with chronic HF	-Increase exercise tolerance, -Pimobendan increased mortality	[21]
**Levosimendan**	RUSSLAN 2002	Randomised, double-blind, placebo-controlled study	(504) Patients with LV failure complicating AMI	To evaluate the safety and efficacy of levosimendan in patients with left ventricular failure complicating acute myocardial infarction	-Low-dose levosimendan reduced the risk of worsening HF	[32]
	LIDO 2002	Multicentre, randomised, double-blind, double-dummy, parallel-group trial	(203) Patients with ADHF	To evaluate the effects of levosimendan vs. dobutamine on haemodynamic performance and clinical outcome in patients with low-output HF	-Improved haemodynamic performance more effectively than dobutamine -Reduced mortality with levosimendan for up to 180 days	[33]
	NCT00048425 REVIVE I and II 2004,2013	Randomised, multicentre, double blind, 2 sequential trials	(700) Patients with ADHF	To evaluate efficacy of iv levosimendan vs. placebo in the short-term treatment of decompensated chronic heart failure	-Rapid and durable symptomatic relief -Increased risk of adverse cardiovascular events and 14-day mortality	[31]
	NCT00348504 SURVIVE 2007	Randomised, double-blind, multicentre, Parallel-group study	(1327) Patients with ADHF	To assess the effect of a short-term IV infusion of levosimendan or dobutamine on long-term survival	-Initial reduction in BNP -No significant reduction of all-cause mortality at 180 days -No effect on any secondary clinical outcomes	[34]

LVEF, left ventricular ejection fraction; HF, heart failure; LV, left ventricle; AMI, acute myocardial infarction; ADHF, acute decompensated heart failure; IV, intravenous; BNP, B-type natriuretic peptide.

**Table 2:** Overview of all reported clinical trials on cardiac myosin activators.

Drug Name	Trial, (Phase) and Year(s)	Study Design	(*n*) Targeted Population	Dose and (Trial Duration)	Primary Endpoint/or Aim	Key Findings	Ref.
**Omecamtiv Mecarbil (formerly CK-1827452 OR AMG 423)**	NCT01380223 (I) 2005–2006	Double-blind, randomised, four-way crossover, placebo-controlled, dose-escalation, single-centre study	(34) Healthy males	IV 0.005–1.0 mg/kg/h (6 h)	To determine maximum tolerated dose of OM	-OM increases SET, SEF, SV, FS (all *p* < 0·0001) -Maximum tolerated dose of OM was 0·5 mg/kg/h	[47]
	NCT00624442 (II) 2007–2009	Double-blind, randomised, placebo controlled, dose-escalation, multicentre international study	(45) Patients with stable chronic systolic heart failure	IV Loading 0.125–1.0 mg/kg/h; maintenance 0.0625–0.5 mg/kg/h (4 treatments at least 7 days apart)	To assess safety and tolerability of OM	-OM caused a concentration-dependent increases in SET and SV, also a reduction in HR was reported (*p* < 0.0001) -Cardiac ischaemia was observed in two patients at high plasma concentrations (» 1750–1350 ng/mL)	[54]
	NCT00682565 (II) 2008	Double-blind, randomised, placebo-controlled, multicentre study	(94) Patients with ischemic cardiomyopathy and angina	IV Loading 24–48 mg/h for 2 h maintenance 6–11 mg/h for 18 h (7 days)	To assess the safety and tolerability of OM during symptom-limited exercise in patients with ischemic cardiomyopathy and angina	-Asymptomatic elevation in troponin and CPK-MB levels	[55]
	NCT01300013 (IIb) ATOMIC-AHF 2013–2015	Double-blind, randomised, placebo-controlled, multicentre Study	(613) Patients with acute systolic heart failure (AHF)	IV Loading 7.5–20 mg/h for 4 h maintenance 1.5–4 mg/h for 44 h (48 h)	Dyspnea relief in patients assessed after 6, 24 and 48 h (using the 7-point Likert scale)	-No improvement in primary endpoint or secondary outcomes -Similar rates of adverse events between treatment and placebo groups -OM increased SET and decreased LVESD	[48]
	NCT01786512 (IIb) COSMIC-HF 2011–2015	Double-blind, randomised, placebo-controlled, multicentre, dose-escalation study	(448) Patients with systemic chronic heart failure with LVEF ≤ 40%	Oral 25 mg twice daily or PK-guided titration to 50 mg twice Daily (20 weeks)	To assess safety, tolerability and pharmacokinetics of OM in 20 weeks of treatment	- OM increased SET and SV - OM reduced HR and NT-proBNP - Comparable adverse events between the groups	[56,57]
	NCT02929329 (III) GALACTIC-HF 2020	Double-blind, randomised, placebo-controlled, multicentre international study	(8256) Patients with symptomatic chronic HF with EF ≤ 35%	Oral 25 mg twice daily or PK-guided titration to 50 mg twice Daily in addition to standard HF therapy (21.8 months)	Time to the next cardiovascular death or first HF event whichever occurred first	-Primary-outcome event occurred in 37% of the OM group and in 39.1% of the placebo group (95% CI 0.86 to 0.99; *p* = 0.03) -10% reduction in the median NT-proBNP level in OM group than placebo group at week 24 compared to Baseline; the median cardiac troponin I level was 4 ng/L higher than baseline.	[50,58,59]
	NCT03759392 (III) METEORIC-HF 2021	Double-blind, randomised, placebo-controlled, multicentre study	(270) Patients with chronic HFrEF	Oral 25 mg twice daily Or PK-guided titration to 50 mg twice Daily (20 weeks)	Change in pVO_2_ on cardiopulmonary exercise testing from baseline to Week 20	Ongoing phase III trial	[60]
**Danicamtiv (MYK-491)**	NCT03062956 (I) 2017	Randomised, placebo-controlled study of single ascending oral doses	(67) Healthy volunteers	Oral Range 3–550 mg (5 days)	To investigate safety, tolerability, pharmacokinetics and pharmacodynamics of MYK-491	-Dose and concentration dependent increased contractility -Modest increase in SET and SV -The drug was generally well-tolerated in the range of 3 to 550 mg	[52]
	NCT03447990 (IIa) 2018–2019	Randomised, double-blind, placebo-controlled, two-part adaptive design study	(40) Patients with HFrEF	175–550 mg or placebo (9 days then follow-up for a week)	To further investigate safety, PK/PD and tolerability of MYK-491	-50 mg BID achieved steady state concentrations at 2000 to 3500 ng/mL -Dose-dependent increase in LVSV, SET -No reports of cardiac ischemia	[53]

OM, Omecamtiv Mecarbil; IV, intravenous; SET, systolic ejection time; SEF, systolic ejection fraction; SV, stroke volume; HR, heart rate; CPK-MB, cardiac creatinine kinase myocardial band; LVESD, left ventricular end-systolic dimension; AHF, acute heart failure; LVEF, left ventricular ejection fraction; PK, pharmacokinetic; PD, pharmacodynamic; NT-proBNP, *N*-terminal pro-B-type natriuretic peptide; HF, heart failure; HFrEF, heart failure with reduced ejection fraction; BID, twice daily; LVSV, left ventricular stroke volume; pVO_2_ peak oxygen uptake.

**Table 3:** Overview of reported key clinical trials on skeletal muscle activators.

Drug Name	Trial and Year(s)	Study Design	(*n*) Targeted Population	Aim	Key Findings	Ref.
**Tirasemtiv (formerly known as** **CK-2017357)**	NCT01709 149 BENEFIT-ALS 2012–2014	Multi-national, double-blind, randomised, placebo-controlled study	(596) Patients with ALS	To evaluate the safety and effectiveness of CK-2017357 when taken with or without riluzole in patients with ALS	-Primary endpoint was not met. -Mixed results were observed for the secondary endpoints.	[77,78]
	NCT02496767 VITALITY-ALS	Multi-national, double-blind, randomised, placebo-controlled, parallel-group study	(744) Patients with ALS	To confirm and extend results from a large phase IIb trial and maximize tolerability with a slower dose escalation	-Primary and secondary endpoints did not show significant differences. -Dizziness, fatigue, nausea, weight loss, and insomnia occurred more frequently on Tirasemtiv. -Tirasemtiv was poorly tolerated.	[79,80]
	NCT02936635 VIGOR-ALS 2016–2018	Open-label extension study	(280) Patients who Completed VITALITY-ALS	To assess the long-term safety and tolerability of Tirasemtiv in patients with ALS	-No available data	[81]
**Reldesemtiv (CK-2127107)**	NCT03065959 2017	Randomised, double-blinded study	(42) Elderly patients with muscle fatigue	To evaluate the effect of Reldesemtiv in elder patients with muscle fatigue	-Trial terminated	[69]
	NCT03160898 FORTITUDE-ALS 2018–2019	Double-blind, randomised, dose-ranging, placebo-controlled parallel group study	(458) Patients with ALS	To evaluate effect of CK-2127107 vs. placebo on respiratory function and other measures of skeletal muscle function in patients with ALS	-No statistical significance In the primary endpoint of change from baseline in SVC after 12 weeks of treatment -All Reldesemtiv groups had declined SVC and ALSFRS-R less than patients on placebo	[71]

ALS, amyotrophic lateral sclerosis; BNP, B-type natriuretic peptide; SVC, slow vital capacity; ALSFRS-R, ALS Functional Rating Scale.

**Table 4:** Overview of reported clinical trials on cardiac myosin inhibitors.

Drug Name	Trial, (Phase) and Year(s)	Study Design	(*n*) Targeted Population	Dose and (Trial Duration)	Primary Endpoint/or Aim	Key Findings	Ref.
**Mavacamten (formerly known as MYK-461)**	NCT02329184 (I) 2014–2016	Open-label, First-in-human study	(15) Patients with HCM	No available data (28 days)	To assess safety, tolerability, preliminary pharmacokinetics and pharmacodynamics of single ascending oral doses	-No available data	[126]
	NCT02842242 PIONEER-HCM (II) 2016–2017	Open-label, Nonrandomised, Pilot Study	(21) Patients with HOCM with resting LVOT gradients of ≥30 or ≥50 mm Hg of provoked gradient	Cohort A Mava 10–20 mg/day w/o background medications Cohort B Mava 2–5 mg/day with b-blockers allowed (12 weeks)	Change in post-exercise peak LVOT gradient from baseline to week 12	-In cohort A, Mava reduced mean post-exercise LVOT gradient from 103 to 19 mmHg at week 12 (*p* = 0.008), reduced resting LVEF and increased Peak VO_2_ -In cohort B, Mava decreased post-exercise LVOT gradient from 86 to 64 mm Hg (*p* = 0.020), 6% mean change in resting LVEF and elevated peak VO_2_ -Most serious AEs are reduced LVEF at higher plasma concentrations and atrial fibrillation	[118,127,128]
	NCT03496168 PIONEER-OLE (II) 2018–2020	Open-label extension study	(13) Patients with HOCM from PIONEER-HCM	After 6-18 months of PIONEER-HCM, Mava was administered in doses of 5,10 or 15 mg (48 weeks)	Frequency and severity of adverse events and serious adverse events	-Interventricular septal thickness was reduced without changes in posterior wall thickness -AEs were mostly mild and transient in nature, no serious adverse events were reported -Mava reduced resting and post-exercise LVOT	[119]
	NCT 03442764 MAVERICK-HCM (II) 2018–2020	Randomised, double-blind, exploratory, placebo-controlled, multicentre, dose-ranging study	(59) Patients with nHCM	Initial dose 5 mg 1 dose titration at week 6 (2.5, 5, 10 or 15 mg) (16 weeks followed by 8 weeks washout)	To assess the safety and tolerability of Mava in patients with systemic nHCM	-SAEs occurred in 10% of participants on Mava and in 21% participants on placebo, indicating significant no difference. -Reversible reduction in LVEF ≤ 45% -NT-proBNP decreased by 53% in the pooled Mava group versus 1% in the placebo group (*p* = 0.0005), 34% reduction in cardiac troponin I in Mava group (*p* = 0.009)	[120,129,130]
	NCT03470545 EXPLORER-HCM (III) 2018–2020	Multicentre, randomised, double-blind, placebo-controlled parallel-group study	(250) Patients with HOCM	Starting dose 5 mg (30 weeks)	1.5 mL/kg per min or greater increase in pVO_2_ and at least one NYHA class reduction **OR** 3 mL/kg per min or greater pVO_2_ increase without NYHA class worsening	-37% of patients on Mava vs. 17% on placebo met the composite primary endpoint (*p* = 0·0005) -A post-exercise LVOT gradient 50 mmHg was achieved in 74% of patients in Mava group and increased pVO_2_ -complete ablation of all LVOT was achieved in 57%	[121,122]
	NCT03723655 MAVA-LTE (III) 2018–2025	Randomised, long-term safety extension study	(310) Patients who completed MAVERICK-HCM or EXPLORER-HCM	No available data (252 weeks)	Frequency and severity of treatment-emergent adverse events and serious AEs	**Ongoing phase III trial**	[131]
	NCT04349072 VALOR-HCM (III) 2020–2024	Randomised, double-blind, placebo-controlled study	(100) Patients with HOCM who are eligible for septal reduction therapy	No available data (32 weeks)	Septal Reduction Therapy (SRT) Status	**Ongoing phase III trial**	[132]
**CK-274**	NCT03767855 (I) 2018–2020	Double-Blind, randomised, placebo-controlled, multi-part, single and multiple ascending dose study	(115) healthy volunteers	No available data (Up to 29 days)	To assess safety, PK and PD of CK-274	- CK-274 was safe and well tolerated in healthy participants. -No serious AEs and clinically meaningful changes in vital signs, ECGs or laboratory tests were observed -Dose-dependent reduction in LVEF	[133,134,135]
	NCT04219826 REDWOOD-HCM (II) 2020–2021	Multicentre, randomised, double-blind, placebo-controlled, dose-finding study	Patients with HOCM	Cohort A 5–10 mg [ECG guided] Cohort 3 10–30 mg of oral CK-274 (10 weeks of treatment and 4 weeks of washout)	To determine the safety and tolerability of CK-274	Ongoing phase II trial	[136]

OM, Omecamtiv Mecarbil; IV, intravenous; SET, systolic ejection time; SEF, systolic ejection fraction; SV, stroke volume; HR, heart rate; CPK-MB, cardiac creatinine kinase myocardial band; LVESD, left ventricular end-systolic dimension; AHF, acute heart failure; LVEF, left ventricular ejection fraction; PK, pharmacokinetic; PD, pharmacodynamic; HF, heart failure; HFrEF, heart failure with reduced ejection fraction; BID, twice daily; LVSV, left ventricular stroke volume; pVO_2_, peak oxygen uptake; ECG, electrocardiogram; OLE, open-label extension.

## References

[1] World Health Organization (WHO) (2020). Cardiovascular Diseases. https://www.who.int/health-topics/cardiovascular-diseases/.

[2] Seferović P.M., Polovina M., Bauersachs J., Arad M., Gal T.B., Lund L.H., Felix S.B., Arbustini E., Caforio A.L.P., Farmakis D. (2019). Heart failure in cardiomyopathies: A position paper from the Heart Failure Association of the European Society of Cardiology. Eur. J. Heart Fail..

[3] Maron B.J., Jeffrey T.A., Gaetano T., Charles A., Domenico C., Donna A., Moss A.J., Seidman C.E., Young B.J. (2006). Contemporary Definitions and Classification of the Cardiomyopathies. Circulation.

[4] Hershberger R.E., Hedges D.J., Morales A. (2013). Dilated cardiomyopathy: The complexity of a diverse genetic architecture. Nat. Rev. Cardiol..

[5] Elliott P., McKenna W.J. (2004). Hypertrophic cardiomyopathy. Lancet.

[6] Ashrafian H., Redwood C., Blair E., Watkins H. (2003). Hypertrophic cardiomyopathy: A paradigm for myocardial energy depletion. Trends Genet..

[7] Tardiff J.C., Carrier L., Bers D.M., Poggesi C., Ferrantini C., Coppini R., Maier L.S., Ashrafian H., Huke S., Van der Velden J. (2015). Targets for therapy in sarcomeric cardiomyopathies. Cardiovasc. Res..

[8] Poggesi C., Ho C.Y. (2014). Muscle dysfunction in hypertrophic cardiomyopathy: What is needed to move to translation?. J. Muscle Res. Cell Motil..

[9] Sewry C.A., Laitila J.M., Wallgren-Pettersson C. (2019). Nemaline myopathies: A current view. J. Muscle Res. Cell Motil..

[10] Papadaki M., Vikhorev P.G., Marston S.B., Messer A.E. (2015). Uncoupling of myofilament Ca^2+^ sensitivity from troponin I phosphorylation by mutations can be reversed by epigallocatechin-3-gallate. Cardiovasc. Res..

[11] Messer A.E., Marston S.B. (2014). Investigating the role of uncoupling of troponin I phosphorylation from changes in myofibrillar Ca^2+^-sensitivity in the pathogenesis of cardiomyopathy. Front. Physiol..

[12] Metra M., Teerlink J.R. (2017). Heart failure. Lancet.

[13] Hartupee J., Mann D.L. (2016). Neurohormonal activation in heart failure with reduced ejection fraction. Nat. Rev. Cardiol..

[14] Ahmad T., Miller P.E., McCullough M., Desai N.R., Riello R., Psotka M., Böhm M., Allen L.A., Teerlink J.R., Rosano G.M.C. (2019). Why has positive inotropy failed in chronic heart failure? Lessons from prior inotrope trials. Eur. J. Heart Fail..

[15] Ponikowski P., Voors A.A., Anker S.D., Bueno H., Cleland J.G.F., Coats A.J.S., Falk V., González-Juanatey J.R., Harjola V.P., Jankowska E.A. (2019). 2016 ESC Guidelines for the diagnosis and treatment of acute and chronic heart failure. Eur. J. Heart Fail..

[16] Yancy C.W., Jessup M., Bozkurt B., Butler J., Casey D.E., Colvin M.M., Drazner M.H., Filippatos G., Fonarow G.C., Givertz M.M. (2016). 2016 ACC/AHA/HFSA focused update on new pharmacological therapy for heart failure: An update of the 2013 ACCF/AHA guideline for the management of heart failure: A report of the American College of Cardiology/American Heart Association Task Force on Clinic. Circulation.

[17] Papp Z., Édes I., Fruhwald S., De Hert S.G., Salmenperä M., Leppikangas H., Mebazaa A., Landoni G., Grossini E., Caimmi P. (2012). Levosimendan: Molecular mechanisms and clinical implications: Consensus of experts on the mechanisms of action of levosimendan. Int. J. Cardiol..

[18] Li M.X., Robertson I.M., Sykes B.D. (2008). Interaction of cardiac troponin with cardiotonic drugs: A structural perspective. Biochem. Biophys. Res. Commun..

[19] Sorsa T., Heikkinen S., Abbott M.B., Abusamhadneh E., Laakso T., Tilgmann C., Serimaa R., Annila A., Rosevear P.R., Drakenberg T. (2001). Binding of Levosimendan, a Calcium Sensitizer, to Cardiac Troponin C. J. Biol. Chem..

[20] Bokník P., Neumann J., Kaspareit G., Schmitz W., Scholz H., Vahlensieck U., Zimmermann N. (1997). Mechanisms of the contractile effects of levosimendan in the mammalian heart. J. Pharmacol. Exp. Ther..

[21] Lubsen J. (1996). Effect of pimobendan on exercise capacity in patients with heart failure: Main results from the Pimobendan in Congestive Heart Failure (PICO) trial. Heart.

[22] AdisInsight (2009). Pimobendan–AdisInsight. https://adisinsight.springer.com/drugs/800000190.

[23] Takahashi R., Endoh M. (2001). Increase in myofibrillar Ca^2+^ sensitivity induced by UD-CG 212 Cl, an active metabolite of pimobendan, in canine ventricular myocardium. J. Cardiovasc. Pharmacol..

[24] Bowles D., Fry D. (2011). Pimobendan and its use in treating canine congestive heart failure. Compend. Contin. Educ. Vet..

[25] AdisInsight (2000). Bepridil-AdisInsight. https://adisinsight.springer.com/drugs/800014884.

[26] AdisInsight (2010). Senazodan-AdisInsight. https://adisinsight.springer.com/drugs/800000316.

[27] Brixius K., Reicke S., Reuter H., Schwinger R.H.G. (2001). Effects of the Ca^2+^ sensitizers EMD 57033 and CGP 48506 on myocardial contractility and Ca^2+^ transients in human ventricular and atrial myocardium. Z. Kardiol..

[28] Baudenbacher F., Schober T., Pinto J.R., Sidorov V.Y., Hilliard F., Solaro R.J., Potter J.D., Knollmann B.C. (2008). Myofilament Ca^2+^ sensitization causes susceptibility to cardiac arrhythmia in mice. J. Clin. Investig..

[29] AdisInsight (2019). AMG 594-AdisInsight. https://adisinsight.springer.com/drugs/800054180#:~:text=AMG.

[30] Reagan J.D., Hartman J.J., Motani A.S., Sutherland W., Poppe L., Hoagland K., Rock B., Lobenhofer E., Nguyen K.K., Liu Q. The novel myotrope, AMG 594, is a small-molecule cardiac troponin activator that increases cardiac contractility in vitro and in vivo. Proceedings of the Keystone Symposia on Molecular and Cellular Biology.

[31] Packer M., Colucci W., Fisher L., Massie B.M., Teerlink J.R., Young J., Padley R.J., Thakkar R., Delgado-Herrera L., Salon J. (2013). Effect of levosimendan on the short-term clinical course of patients with acutely decompensated heart failure. JACC Heart Fail..

[32] Moiseyev V.S., Põder P., Andrejevs N., Ruda M.Y., Golikov A.P., Lazebnik L.B., Kobalava Z.D., Lehtonen L.A., Laine T., Nieminen M.S. (2002). Safety and efficacy of a novel calcium sensitizer, levosimendan, in patients with left ventricular failure due to an acute myocardial infarction: A randomized, placebo-controlled, double-blind study (RUSSLAN). Eur. Heart J..

[33] Follath F., Cleland J.G.F., Just H., Papp J.G.Y., Scholz H., Peuhkurinen K., Harjola V.P., Mitrovic V., Abdalla M., Sandell E.P. (2002). Efficacy and safety of intravenous levosimendan compared with dobutamine in severe low-output heart failure (the LIDO study): A randomised double-blind trial. Lancet.

[34] Mebazaa A., Nieminen M.S., Packer M., Cohen-Solal A., Kleber F.X., Pocock S.J., Thakkar R., Padley R.J., Põder P., Kivikko M. (2007). Levosimendan vs. dobutamine for patients with acute decompensated heart failure: The SURVIVE randomized trial. J. Am. Med. Assoc..

[35] Malik F.I., Hartman J.J., Elias K.A., Morgan B.P., Rodriguez H., Brejc K., Anderson R.L., Sueoka S.H., Lee K.H., Finer J.T. (2011). Cardiac myosin activation: A potential therapeutic approach for systolic heart failure. Science.

[36] Malik F.I., Morgan B.P. (2011). Cardiac myosin activation part 1: From concept to clinic. J. Mol. Cell Cardiol..

[37] Packer M., Kukin M.L., Sollano J.A., Carver J.R., Rodeheffer R.J., Ivanhoe R.J., Dibianco R., Zeldis S.M., Hendrix G.H., Bommer W.J. (1991). Effect of Oral Milrinone on Mortality in Severe Chronic Heart Failure. N. Engl. J. Med..

[38] O’Connor C.M., Gattis W.A., Uretsky B.F., Adams K.F., McNulty S.E., Grossman S.H., McKenna W.J., Zannad F., Swedberg K., Gheorghiade M. (1999). Continuous intravenous dobutamine is associated with an increased risk of death in patients with advanced heart failure: Insights from the Flolan International Randomized Survival Trial (FIRST). Am. Heart J..

[39] Morgan B.P., Muci A., Lu P.P., Qian X., Tochimoto T., Smith W.W., Garard M., Kraynack E., Collibee S., Suehiro I. (2010). Discovery of omecamtiv mecarbil the first, selective, small molecule activator of cardiac myosin. ACS Med. Chem. Lett..

[40] Swenson A.M., Tang X.W., Blair C.A., Fetrow C.M., Unrath W.C., Previs M.J., Campbell K.S., Yengo C.M. (2017). Omecamtiv mecarbil enhances the duty ratio of human β-cardiac myosin resulting in increased calcium sensitivity and slowed force development in cardiac muscle. J. Biol. Chem..

[41] Nagy L., Kovács A., Bõdi B., Pásztor E.T., Fülöp G., Tõth A., Édes I., Papp Z. (2015). The novel cardiac myosin activator omecamtiv mecarbil increases the calcium sensitivity of force production in isolated cardiomyocytes and skeletal muscle fibres of the rat. Br. J. Pharmacol..

[42] Liu Y., White H.D., Belknap B., Winkelmann D.A., Forgacs E. (2015). Omecamtiv Mecarbil Modulates the Kinetic and Motile Properties of Porcine β-Cardiac Myosin. Biochemistry.

[43] Woody M.S., Greenberg M.J., Barua B., Winkelmann D.A., Goldman Y.E., Ostap E.M. (2018). Positive cardiac inotrope omecamtiv mecarbil activates muscle despite suppressing the myosin working stroke. Nat. Commun..

[44] Lehrer S.S., Geeves M.A. (1998). The muscle thin filament as a classical cooperative/allosteric regulatory system11Edited by P. E. Wright. J. Mol. Biol..

[45] Bakkehaug J.P., Kildal A.B., Engstad E.T., Boardman N., Næsheim T., Rønning L., Aasum E., Larsen T.S., Myrmel T., How O.J. (2015). Myosin activator omecamtiv mecarbil increases myocardial oxygen consumption and impairs cardiac efficiency mediated by resting myosin ATPase activity. Circ. Heart Fail..

[46] Teerlink J.R., Malik F.I., Kass D.A. (2015). Letter by Teerlink et al Regarding Article, “Myosin Activator Omecamtiv Mecarbil Increases Myocardial Oxygen Consumption and Impairs Cardiac Efficiency Mediated by Resting Myosin ATPase Activity”. Circ. Heart Fail..

[47] Teerlink J.R., Clarke C.P., Saikali K.G., Lee J.H., Chen M.M., Escandon R.D., Elliott L., Bee R., Habibzadeh M.R., Goldman J.H. (2011). Dose-dependent augmentation of cardiac systolic function with the selective cardiac myosin activator, omecamtiv mecarbil: A first-in-man study. Lancet.

[48] Teerlink J.R., Felker G.M., McMurray J.J.V., Ponikowski P., Metra M., Filippatos G.S., Ezekowitz J.A., Dickstein K., Cleland J.G.F., Kim J.B. (2016). Acute Treatment with Omecamtiv Mecarbil to Increase Contractility in Acute Heart Failure: The ATOMIC-AHF Study. J. Am. Coll. Cardiol..

[49] Starling R.C. (2016). Cardiac Myosin Activators for the Treatment of Heart Failure: Stop Now or Push Ahead?. J. Am. Coll. Cardiol..

[50] Teerlink J.R., Diaz R., Felker G.M., McMurray J.J.V.V., Metra M., Solomon S.D., Adams K.F., Anand I., Arias-Mendoza A., Biering-Sørensen T. (2020). Cardiac Myosin Activation with Omecamtiv Mecarbil in Systolic Heart Failure. N. Engl. J. Med..

[51] Fernandes S., Oikonomopoulos A., Jimenez-MacInnes S.K., Aschar-Sobbi R., Henze M., Sumandea M., Gan Q.F., Anderson R.L., Del Rio C.L. (2019). MYK-491, a Novel Small-Molecule Cardiac Myosin Activator Increases Cardiac Systolic Function and Preserves Mechanical Efficiency: Pre-Clinical in vivo and in vitro Evidence. Circulation.

[52] Tamby J.F., Fang L., Lickliter J., Hegde S., Surks H., Reele S., Teichman S., Yang C., Fernandes S., Lambing J. (2019). MYK-491, a Novel Cardiac Myosin Activator, Increases Cardiac Contractility in Healthy Volunteers. Eur. J. Heart Fail..

[53] Voors A.A., Tamby J.F., Cleland J.G., Koren M., Forgosh L.B., Gupta D., Lund L.H., Camacho A., Karra R., Swart H.P. (2020). Effects of danicamtiv, a novel cardiac myosin activator, in heart failure with reduced ejection fraction: Experimental data and clinical results from a phase 2a trial. Eur. J. Heart Fail..

[54] Cleland J.G.F., Teerlink J.R., Senior R., Nifontov E.M., Mc Murray J.J.V., Lang C.C., Tsyrlin V.A., Greenberg B.H., Mayet J., Francis D.P. (2011). The effects of the cardiac myosin activator, omecamtiv mecarbil, on cardiac function in systolic heart failure: A double-blind, placebo-controlled, crossover, dose-ranging phase 2 trial. Lancet.

[55] Greenberg B.H., Chou W., Saikali K.G., Escandón R., Lee J.H., Chen M.M., Treshkur T., Megreladze I., Wasserman S.M., Eisenberg P. (2015). Safety and Tolerability of Omecamtiv Mecarbil During Exercise in Patients with Ischemic Cardiomyopathy and Angina. JACC Heart Fail..

[56] Teerlink J.R., Felker G.M., McMurray J.J.V., Solomon S.D., Adams K.F., Cleland J.G.F., Ezekowitz J.A., Goudev A., Macdonald P., Metra M. (2016). Chronic Oral Study of Myosin Activation to Increase Contractility in Heart Failure (COSMIC-HF): A phase 2, pharmacokinetic, randomised, placebo-controlled trial. Lancet.

[57] Teerlink J.R., Felker G.M., McMurray J., Solomon S., Cleland J., Goldsmith S., Kurtz C., Buchele G., Legg J., Malik F. (2019). Effect of Omecamtiv Mecarbil in Patients With Atrial Fibrillation and Heart Failure With Reduced Ejection Fraction: Results From Cosmic-Hf. J. Am. Coll. Cardiol..

[58] Teerlink J.R., Diaz R., Felker G.M., McMurray J.J.V., Metra M., Solomon S.D., Legg J.C., Büchele G., Varin C., Kurtz C.E. (2020). Omecamtiv Mecarbil in Chronic Heart Failure With Reduced Ejection Fraction: Rationale and Design of GALACTIC-HF. JACC Heart Fail..

[59] Teerlink J.R., Diaz R., Felker G.M., McMurray J.J.V., Metra M., Solomon S.D., Legg J.C., Büchele G., Varin C., Kurtz C.E. (2020). Baseline characteristics from the cardiovascular outcomes trial of omecamtiv mecarbil (GALACTIC-HF). J. Am. Coll. Cardiol..

[60] Lewis G., Böhm M., Cohen-Solal A., Ezekowitz J., Metra M., Ponikowski P., Teerlink J., Voors A., Whellan D., Legg J. Multicenter Exercise Tolerance Evaluation of Omecamtiv Mecarbil Related to Increased Contractility in Heart Failure (METEORIC-HF). Proceedings of the Heart Failure Society of America 23rd Annual Scientific Meeting.

[61] Radke M.B., Taft M.H., Stapel B., Hilfiker-Kleiner D., Preller M., Manstein D.J. (2014). Small molecule-mediated refolding and activation of myosin motor function. eLife.

[62] Amgen FDA Grants Fast Track Designation for Omecamtiv Mecarbil in Heart Failure. https://www.amgen.com/media/news-releases/2020/05/fda-grants-fast-track-designation-for-omecamtiv-mecarbil-in-heart-failure/.

[63] Silber E.N., Katz L.N. (2000). Therapeutic strategies for managing heart failure. Heart Failure: Pathophysiology, Molecular Biology, and Clinical Management.

[64] Russell A.J., Hartman J.J., Hinken A.C., Muci A.R., Kawas R., Driscoll L., Godinez G., Lee K.H., Marquez D., Browne Iv W.F. (2012). Activation of fast skeletal muscle troponin as a potential therapeutic approach for treating neuromuscular diseases. Nat. Med..

[65] Marston S. (2019). Small molecule studies: The fourth wave of muscle research. J. Muscle Res. Cell Motil..

[66] Hansen R., Saikali K.G., Chou W., Russell A.J., Chen M.M., Vijayakumar V., Stoltz R.R., Baudry S., Enoka R.M., Morgans D.J. (2014). Tirasemtiv amplifies skeletal muscle response to nerve activation in humans. Muscle Nerve.

[67] Cytokinetics. Cytokinetics Announces Orphan Drug Designation Granted to CK-2017357 for the Treatment of Amyotrophic Lateral Sclerosis | Cytokinetics, Inc. http://ir.cytokinetics.com/news-releases/news-release-details/cytokinetics-announces-orphan-drug-designation-granted-ck.

[68] Cytokinetics. Cytokinetics Announces Negative Results From VITALITY-ALS|Cytokinetics, Inc. http://ir.cytokinetics.com/news-releases/news-release-details/cytokinetics-announces-negative-results-vitality-als.

[69] AdisInsight (2013). Reldesemtiv-Astellas Pharma/Cytokinetics-AdisInsight. https://adisinsight.springer.com/drugs/800037810.

[70] Andrews J.A., Miller T.M., Vijayakumar V., Stoltz R., James J.K., Meng L., Wolff A.A., Malik F.I. (2018). CK-2127107 amplifies skeletal muscle response to nerve activation in humans. Muscle Nerve.

[71] CytoKinetics. Cytokinetics Announces Results of FORTITUDE-ALS, a Phase 2 Clinical Trial of Reldesemtiv in Patients With ALS, Presented at American Academy of Neurology Annual Meeting | Cytokinetics, Inc. http://ir.cytokinetics.com/news-releases/news-release-details/cytokinetics-announces-results-fortitude-als-phase-2-clinical.

[72] Cheng A.J., Hwee D.T., Kim L.H., Durham N., Yang H.T., Hinken A.C., Kennedy A.R., Terjung R.L., Jasper J.R., Malik F.I. (2019). Fast skeletal muscle troponin activator CK-2066260 increases fatigue resistance by reducing the energetic cost of muscle contraction. J. Physiol..

[73] Lee E.J., De Winter J.M., Buck D., Jasper J.R., Malik F.I., Labeit S., Ottenheijm C.A., Granzier H. (2013). Fast Skeletal Muscle Troponin Activation Increases Force of Mouse Fast Skeletal Muscle and Ameliorates Weakness Due to Nebulin-Deficiency. PLoS ONE.

[74] Nogara L., Naber N., Pate E., Canton M., Reggiani C., Cooke R. (2016). Piperine’s mitigation of obesity and diabetes can be explained by its up-regulation of the metabolic rate of resting muscle. Proc. Natl. Acad. Sci. USA.

[75] Cooke R. (2011). The role of the myosin ATPase activity in adaptive thermogenesis by skeletal muscle. Biophys. Rev..

[76] Tiwari A., Mahadik K.R., Gabhe S.Y. (2020). Piperine: A comprehensive review of methods of isolation, purification, and biological properties. Med. Drug Discov..

[77] Shefner J.M., Wolff A.A., Meng L., Bian A., Lee J., Barragan D., Andrews J.A. (2016). A randomized, placebo-controlled, double-blind phase IIb trial evaluating the safety and efficacy of tirasemtiv in patients with amyotrophic lateral sclerosis. Amyotroph. Later. Scler. Frontotempo. Degener..

[78] (2014). Cytokinetics. Cytokinetics Announces Top-Line Results From BENEFIT-ALS|Cytokinetics, Inc. https://adisinsight.springer.com/trials/700221015.

[79] Andrews J.A., Cudkowicz M.E., Hardiman O., Meng L., Bian A., Lee J., Wolff A.A., Malik F.I., Shefner J.M. (2018). VITALITY-ALS, a phase III trial of tirasemtiv, a selective fast skeletal muscle troponin activator, as a potential treatment for patients with amyotrophic lateral sclerosis: Study design and baseline characteristics. Amyotroph. Later. Scler. Frontotempo. Degener..

[80] Shefner J.M., Cudkowicz M.E., Hardiman O., Cockroft B.M., Lee J.H., Malik F.I., Meng L., Rudnicki S.A., Wolff A.A., Andrews J.A. (2019). A phase III trial of *Tirasemtiv* as a potential treatment for amyotrophic lateral sclerosis. Amyotroph. Later. Scler. Frontotempo. Degener..

[81] AdisInsight (2009). Tirasemtiv-Cytokinetics–AdisInsight. https://adisinsight.springer.com/drugs/800030378.

[82] Spudich J.A. (2015). The myosin mesa and a possible unifying hypothesis for the molecular basis of human hypertrophic cardiomyopathy. Biochem. Soc. Trans..

[83] Spudich J.A. (2019). Three perspectives on the molecular basis of hypercontractility caused by hypertrophic cardiomyopathy mutations. Pflug. Arch. Eur. J. Physiol..

[84] Buvoli M., Hamady M., Leinwand L.A., Knight R. (2008). Bioinformatics Assessment of β-Myosin Mutations Reveals Myosin’s High Sensitivity to Mutations. Trends Cardiovasc. Med..

[85] Walsh R., Rutland C., Thomas R., Loughna S. (2009). Cardiomyopathy: A systematic review of disease-causing mutations in myosin heavy chain 7 and their phenotypic manifestations. Cardiology.

[86] Spudich J.A. (2014). Hypertrophic and Dilated Cardiomyopathy: Four Decades of Basic Research on Muscle Lead to Potential Therapeutic Approaches to These Devastating Genetic Diseases. Biophys. J..

[87] Gersh B.J., Maron B.J., Bonow R.O., Dearani J.A., Fifer M.A., Link M.S., Naidu S.S., Nishimura R.A., Ommen S.R., Rakowski H. (2011). 2011 ACCF/AHA guideline for the diagnosis and treatment of hypertrophic cardiomyopathy: A report of the American College of cardiology foundation/American heart association task force on practice guidelines. Circulation.

[88] Zamorano J.L., Anastasakis A., Borger M.A., Borggrefe M., Cecchi F., Charron P., Hagege A.A., Lafont A., Limongelli G., Mahrholdt H. (2014). 2014 ESC guidelines on diagnosis and management of hypertrophic cardiomyopathy: The task force for the diagnosis and management of hypertrophic cardiomyopathy of the European Society of Cardiology (ESC). Eur. Heart J..

[89] Arts I.C.W., Hollman P.C.H., Feskens E.J.M., Bueno de Mesquita H.B., Kromhout D. (2001). Catechin intake might explain the inverse relation between tea consumption and ischemic heart disease: The Zutphen Elderly Study. Am. J. Clin. Nutr..

[90] Kokubo Y., Iso H., Saito I., Yamagishi K., Yatsuya H., Ishihara J., Inoue M., Tsugane S. (2013). The impact of green tea and coffee consumption on the reduced risk of stroke incidence in japanese population: The Japan public health center-based study cohort. Stroke.

[91] Harbowy M.E., Balentine D.A., Davies A.P., Cai Y. (1997). Tea Chemistry. Crit. Rev. Plant Sci..

[92] Tadano N., Du C.K., Yumoto F., Morimoto S., Ohta M., Xie M.F., Nagata K., Zhan D.Y., Lu Q.W., Miwa Y. (2010). Biological actions of green tea catechins on cardiac troponin C. Br. J. Pharmacol..

[93] Robinson P.J., Patel S., Liu X., Zhang Y.-H., Khandelwal A., Blagg B., Casadei B., Watkins H., Redwood C. (2016). Novel Potential Treatment of Familial Hypertrophic Cardiomyopathy with Analogues of the Green Tea Polyphenol Epigallocatechin-3-Gallate. Biophys. J..

[94] Zeitz O., Rahman A., Hasenfuss G., Janssen P.M.L. (2000). Impact of β-adrenoceptor antagonists on myofilament calcium sensitivity of rabbit and human myocardium. J. Cardiovas. Pharmacol..

[95] Stücker S., Kresin N., Carrier L., Friedrich F.W. (2017). Nebivolol desensitizes myofilaments of a hypertrophic cardiomyopathy mouse model. Front. Physiol..

[96] Wright P.T., Tsui S.F., Francis A.J., MacLeod K.T., Marston S.B. (2020). Approaches to High-Throughput Analysis of Cardiomyocyte Contractility. Front. Physiol..

[97] Sheehan A., Messer A.E., Papadaki M., Choudhry A., Kren V., Biedermann D., Blagg B., Khandelwal A., Marston S.B. (2018). Molecular defects in cardiac myofilament Ca^2+^-regulation due to cardiomyopathy-linked mutations can be reversed by small molecules binding to troponin. Front. Physiol..

[98] Baell J., Walters M.A. (2014). Chemical con artists foil drug discovery. Nature.

[99] Ingólfsson H.I., Thakur P., Herold K.F., Hobart E.A., Ramsey N.B., Periole X., De Jong D.H., Zwama M., Yilmaz D., Hall K. (2014). Phytochemicals perturb membranes and promiscuously alter protein function. ACS Chem. Biol..

[100] Memo M., Leung M.C., Ward D.G., Dos Remedios C., Morimoto S., Zhang L., Ravenscroft G., McNamara E., Nowak K.J., Marston S.B. (2013). Familial dilated cardiomyopathy mutations uncouple troponin I phosphorylation from changes in myofibrillar Ca2+ sensitivity. Cardiovasc. Res..

[101] Vikhorev P.G., Song W., Wilkinson R., Copeland O.N., Messer A.E., Ferenczi M.A., Marston S.B. (2014). The dilated cardiomyopathy-causing mutation ACTC E361G in cardiac muscle myofibrils specifically abolishes modulation of Ca^2+^ regulation by phosphorylation of troponin I. Biophys. J..

[102] Wilkinson R., Song W., Smoktunowicz N., Marston S. (2015). A dilated cardiomyopathy mutation blunts adrenergic response and induces contractile dysfunction under chronic angiotensin II stress. Am. J. Physiol. Circ. Physiol..

[103] Wilson I.B., Ginsburg S. (1955). A powerful reactivator of alkylphosphate-inhibited acetylcholinesterase. BBA Biochim. Biophys. Acta.

[104] Cheung A., Dantzig J.A., Hollingworth S., Baylor S.M., Goldman Y.E., Mitchinson T.J., Straight A.F. (2002). A small-molecule inhibitor of skeletal muscle myosin II. Nat. Cell Biol..

[105] Straight A.F., Cheung A., Limouze J., Chen I., Westwood N.J., Sellers J.R., Mitchison T.J. (2003). Dissecting temporal and spatial control of cytokinesis with a myosin II inhibitor. Science.

[106] Abi-Gerges N., Pointon A., Pullen G.F., Morton M.J., Oldman K.L., Armstrong D., Valentin J.P., Pollard C.E. (2013). Preservation of cardiomyocytes from the adult heart. J. Mol. Cell Cardiol..

[107] Rauscher A., Gyimesi M., Kovács M., Málnási-Csizmadia A. (2018). Targeting Myosin by Blebbistatin Derivatives: Optimization and Pharmacological Potential. Trends Biochem. Sci..

[108] Wilson C., Naber N., Pate E., Cooke R. (2014). The myosin inhibitor blebbistatin stabilizes the super-relaxed state in skeletal muscle. Biophys. J..

[109] Roman B.I., Verhasselt S., Stevens C.V. (2018). Medicinal Chemistry and Use of Myosin II Inhibitor (S)-Blebbistatin and Its Derivatives. J. Med. Chem..

[110] Spudich J.A., Aksel T., Bartholomew S.R., Nag S., Kawana M., Yu E.C., Sarkar S.S., Sung J., Sommese R.F., Sutton S. (2016). Effects of hypertrophic and dilated cardiomyopathy mutations on power output by human β-cardiac myosin. J. Exp. Biol..

[111] Tekakirikul P., Eminaga S., Toka O., Alcalai R., Wang L., Wakimoto H., Nayor M., Konno T., Gorham J.M., Wolf C.M. (2010). Cardiac fibrosis in mice with hypertrophic cardiomyopathy is mediated by non-myocyte proliferation and requires Tgf-β. J. Clin. Investig..

[112] Green E.M., Wakimoto H., Anderson R.L., Evanchik M.J., Gorham J.M., Harrison B.C., Henze M., Kawas R., Oslob J.D., Rodriguez H.M. (2016). Heart disease: A small-molecule inhibitor of sarcomere contractility suppresses hypertrophic cardiomyopathy in mice. Science.

[113] Anderson R.L., Trivedi D.V., Sarkar S.S., Henze M., Ma W., Gong H., Rogers C.S., Gorham J.M., Wong F.L., Morck M.M. (2018). Deciphering the super relaxed state of human β-cardiac myosin and the mode of action of mavacamten from myosin molecules to muscle fibers. Proc. Natl. Acad. Sci. USA.

[114] Rohde J.A., Roopnarine O., Thomas D.D., Muretta J.M. (2018). Mavacamten stabilizes an autoinhibited state of two-headed cardiac myosin. Proc. Natl. Acad. Sci. USA.

[115] Stern J.A., Markova S., Ueda Y., Kim J.B., Pascoe P.J., Evanchik M.J., Green E.M., Harris S.P. (2016). A small molecule inhibitor of sarcomere contractility acutely relieves left ventricular outflow tract obstruction in feline hypertrophic cardiomyopathy. PLoS ONE.

[116] Del Rio C.L., Yukie U., Baker D.C., Dalton R.S., Laurence L., Philip J., Bari O., Joseph L., Evanchik M.J., Green E.M. (2017). Abstract 20593: In vivo Cardiac Effects of Mavacamten (MYK-461): Evidence for Negative Inotropy and Improved Compliance. Circulation.

[117] Grillo M.P., Erve J.C.L.L., Dick R., Driscoll J.P., Haste N., Markova S., Brun P., Carlson T.J., Evanchik M. (2019). In vitro and in vivo pharmacokinetic characterization of mavacamten, a first-in-class small molecule allosteric modulator of beta cardiac myosin. Xenobiotica.

[118] Heitner S.B., Jacoby D., Lester S.J., Owens A., Wang A., Zhang D., Lambing J., Lee J., Semigran M., Sehnert A.J. (2019). Mavacamten treatment for obstructive hypertrophic cardiomyopathy a clinical trial. Ann. Intern. Med..

[119] Heitner S.B., Lester S., Wang A., Hegde S.M., Fang L., Balaratnam G., Sehnert A.J., Jacoby D. (2019). Precision Pharmacological Treatment for Obstructive Hypertrophic Cardiomyopathy With Mavacamten: One-Year Results From PIONEER-OLE. Circulation.

[120] Ho C.Y., Mealiffe M.E., Bach R.G., Bhattacharya M., Choudhury L., Edelberg J.M., Hegde S.M., Jacoby D., Lakdawala N.K., Lester S.J. (2020). Evaluation of Mavacamten in Symptomatic Patients With Nonobstructive Hypertrophic Cardiomyopathy. J. Am. Coll. Cardiol..

[121] Olivotto I., Oreziak A., Barriales-villa R., Abraham T.P., Masri A., Garcia-pavia P., Saberi S., Lakdawala N.K., Hegde S.M., Solomon S.D. (2020). Mavacamten for treatment of symptomatic obstructive hypertrophic cardiomyopathy (EXPLORER-HCM): A randomised, double-blind, placebo-controlled, phase 3 trial. Lancet.

[122] Ho C.Y., Olivotto I., Jacoby D., Lester S.J., Roe M., Wang A., Waldman C.B., Zhang D., Sehnert A.J., Heitner S.B. (2020). Study Design and Rationale of EXPLORER-HCM: Evaluation of Mavacamten in Adults with Symptomatic Obstructive Hypertrophic Cardiomyopathy. Circ. Heart Fail..

[123] Del Rio C.L., Aprajita Y., S F.B., Christopher Z., Trisha S., Frank R., John S., Lee-Jae G., Allison H., Julie G. (2019). Abstract 14585: Chronic Treatment With a Mavacamten-Like Myosin-Modulator (MYK-581) Blunts Disease Progression in a Mini-Pig Genetic Model of Non-Obstructed Hypertrophic Cardiomyopathy: In Vivo Evidence for Improved Relaxation and Functional Reserve. Circulation.

[124] (2020). MyoKardia Programs|MyoKardia. https://myokardia.com/programs.

[125] AdisInsight A Phase 1 Randomized, Placebo-Controlled, Single and Multiple-Ascending Dose Study of MYK-224 in Healthy Volunteers—AdisInsight. https://adisinsight.springer.com/trials/700301316.

[126] NIH Study Evaluating the Safety, Tolerability and Preliminary Pharmacokinetics and Pharmacodynamics of MYK-461-Full Text View—ClinicalTrials.gov. https://clinicaltrials.gov/ct2/show/NCT02329184.

[127] Jacoby D., Lester S., Owens A., Wang A., Young D., Tripuraneni R., Semigran M., Heitner S. (2018). Reduction in left ventricular outflow tract gradient with mavacamten (myk-461) in symptomatic obstructive hypertrophic cardiomyopathy patients (PIONEER-HCM). J. Am. Coll. Cardiol..

[128] Heitner S.B., Jacoby D., Lester S., Owens A.T., Wang A., Shah A., Hegde S., Fang L., Sehnert A.J., Semigran M. (2018). Mavacamten Improves Left Ventricular Relaxation and Compliance in Obstructive Hypertrophic Cardiomyopathy Through Direct Myosin Modulation. Circulation.

[129] Ho C.Y. Safety and Efficacy of Mavacamten in Symptomatic Non-Obstructive Hypertrophic Cardiomyopathy: The MAVERICK-HCM Study. Proceedings of the American College of Cardiology (ACC)/World Congress of Cardiology’s (WCC) Virtual Scientific Sessions.

[130] Heitner S., Wang A., Jacoby D., Lester S., Carlson T., Zhang D., Sehnert A., Ho C. (2018). Maverick-HCM: Phase 2 randomized, multi-center, double-blind, placebo-controlled concentration-guided study to evaluate mavacamten (MYK-461) in adults with symptomatic non-obstructive hypertrophic cardiomyopathy. Circulation.

[131] NIH A Long-Term Safety Extension Study of Mavacamten in Adults Who Have Completed MAVERICK-HCM or EXPLORER-HCM—Full Text View—ClinicalTrials.gov. https://clinicaltrials.gov/ct2/show/NCT03723655.

[132] NIH A Study to Evaluate Mavacamten in Adults With Symptomatic Obstructive HCM Who Are Eligible for Septal Reduction Therapy—Full Text View—ClinicalTrials.gov. https://clinicaltrials.gov/ct2/show/NCT04349072.

[133] NIH A Single and Multiple Ascending Dose Study of CK-3773274 in Healthy Adult Subjects—Full Text View—ClinicalTrials.gov. https://www.clinicaltrials.gov/ct2/show/NCT03767855?term=ck-3773274.

[134] CytoKinetics. CytoKinetics Announces Data from Phase 1 Study of CK- 3773274 at the HFSA 23rd Annual Scientific Meeting. http://ir.cytokinetics.com/news-releases/news-release-details/cytokinetics-announces-data-phase-1-study-ck-3773274-hfsa-23r.

[135] Robertson L.A., Armas D.R., Robbie E., Osmukhina A., Li H., Malik F.I., Solomon S.D. (2019). A First in Human Study of the Selective Cardiac Myosin Inhibitor, CK-3773274. J. Card. Fail..

[136] NIH REDWOOD-HCM: Randomized Evaluation of Dosing with CK-3773274 in Obstructive Outflow Disease in HCM—Full Text View—ClinicalTrials.gov. https://clinicaltrials.gov/ct2/show/NCT04219826.

[137] Hartman J.J., Hwee D.T., Wang J., Wu Y., Schaletzky J., Paliwal P., Lee K., Taheri K.D., Wehri E., Ewing T.J. (2020). Characterization of the Cardiac Myosin Inhibitor CK-3773274: A Potential Therapeutic Approach for Hypertrophic Cardiomyopathy. Biophys. J..

[138] Hwee D.T., Hartman J.J., Wang J., Wu Y., Schaletzky J., Paliwal P., Lee K., Taheri K.D., Wehri E., Chuang C. (2019). Pharmacologic Characterization of the Cardiac Myosin Inhibitor, CK-3773274: A Potential Therapeutic Approach for Hypertrophic Cardiomyopathy. Circ. Res..

[139] Hwee D.T., Wu Y., Cremin P., Morgan B.P., Malik F.I., Chin E.R. (2019). The Cardiac Myosin Inhibitor, CK-3773274, Reduces Contractility in the R403q Mouse Model of Hypertrophic Cardiomyopathy. Circ. Res..

[140] AdisInsight (2020). CK 274—AdisInsight. https://adisinsight.springer.com/drugs/800053181.

[141] Myokardia. MyoKardia Announces Receipt of Breakthrough Therapy Designation from FDA for Mavacamten for the Treatment of Symptomatic, Obstructive Hypertrophic Cardiomyopathy Nasdaq:MYOK. https://www.globenewswire.com/news-release/2020/07/23/2066908/0/en/MyoKardia-Announces-Receipt-of-Breakthrough-Therapy-Designation-from-FDA-for-Mavacamten-for-the-Treatment-of-Symptomatic-Obstructive-Hypertrophic-Cardiomyopathy.html.

[142] Ashrafian H., Frenneaux M.P., Opie L.H. (2007). Metabolic mechanisms in heart failure. Circulation.

[143] MyoKardia. MyoKardia Announces Positive Topline Data from its Phase 2 MAVERICK-HCM Clinical Trial of Mavacamten|Bristol-Myers Squibb. http://investors.myokardia.com/news-releases/news-release-details/myokardia-announces-positive-topline-data-its-phase-2-maverick.

